# Evolution of KaiC-Dependent Timekeepers: A Proto-circadian Timing Mechanism Confers Adaptive Fitness in the Purple Bacterium *Rhodopseudomonas palustris*

**DOI:** 10.1371/journal.pgen.1005922

**Published:** 2016-03-16

**Authors:** Peijun Ma, Tetsuya Mori, Chi Zhao, Teresa Thiel, Carl Hirschie Johnson

**Affiliations:** 1 Department of Biological Sciences, Vanderbilt University, Nashville, Tennessee, United States of America; 2 Department of Biology, University of Missouri-St. Louis, St. Louis, Missouri, United States of America; Max Planck Institute for Terrestrial Microbiology, GERMANY

## Abstract

Circadian (daily) rhythms are a fundamental and ubiquitous property of eukaryotic organisms. However, cyanobacteria are the only prokaryotic group for which *bona fide* circadian properties have been persuasively documented, even though homologs of the cyanobacterial *kaiABC* central clock genes are distributed widely among Eubacteria and Archaea. We report the purple non-sulfur bacterium *Rhodopseudomonas palustris (*that harbors homologs of *kaiB* and *kaiC*) only poorly sustains rhythmicity in constant conditions–a defining characteristic of circadian rhythms. Moreover, the biochemical characteristics of the *Rhodopseudomonas* homolog of the KaiC protein *in vivo* and *in vitro* are different from those of cyanobacterial KaiC. Nevertheless, *R*. *palustris* cells exhibit adaptive *kaiC*-dependent growth enhancement in 24-h cyclic environments, but not under non-natural constant conditions. Therefore, our data indicate that *Rhodopseudomonas* does not have a classical circadian rhythm, but a novel timekeeping mechanism that does not sustain itself in constant conditions. These results question the adaptive value of self-sustained oscillatory capability for daily timekeepers and establish new criteria for circadian-like systems that are based on adaptive properties (i.e., fitness enhancement in rhythmic environments), rather than upon observations of persisting rhythms in constant conditions. We propose that the *Rhodopseudomonas* system is a "proto" circadian timekeeper, as in an ancestral system that is based on KaiC and KaiB proteins and includes some, but not necessarily all, of the canonical properties of circadian clocks. These data indicate reasonable intermediate steps by which *bona fide* circadian systems evolved in simple organisms.

## Author Summary

Circadian (daily) rhythms are an adaptation of organisms to the dramatic changes in the environment over day and night, and they are defined by properties that include rhythmic persistence in constant conditions and temperature compensation. These "biological clocks" are practically ubiquitous in eukaryotes, but in prokaryotes they have been persuasively documented only among cyanobacterial species, where the *kaiABC* genes regulate a biochemical timekeeping mechanism. The *kaiB* and *kaiC* genes are found widely among other prokaryotic species, but non-cyanobacterial prokaryotes usually have poor persistence of rhythmic function in constant conditions, and are therefore considered to not have circadian systems. We have examined a purple non-sulfur bacterium (*Rhodopseudomonas palustris*) that harbors *kaiB* and *kaiC* homologs. Its daily rhythm of nitrogen fixation does not continue in constant conditions, and biochemical properties of the KaiC protein that are known to be crucial for circadian rhythmicity in cyanobacteria are altered in *Rhodopseudomonas'* KaiC. Nevertheless, we find that adaptive growth of the cells is dependent upon *kaiC* in rhythmic environments, but not in non-rhythmic environments. We propose that the *Rhodopseudomonas* system is an ancestral "proto-circadian" timekeeper that is based on KaiC and KaiB, and that elucidates intermediate steps by which biological clocks evolved in simple organisms.

## Introduction

Circadian (daily) rhythms are a fundamental property of cellular and organismal organization that regulate gene expression, metabolic activity, sleep, behavior, and many other biological processes to provide a fitness advantage [1–4]. These rhythms have three defining and "canonical" characteristics. The first is sustained persistence in constant conditions with a period of ~ 24 h. The second is the establishment of a particular phase relationship (aka “phase angle”) between the endogenous biological clock and the daily environmental cycle by entrainment. The final characteristic is that the period of the free-running rhythm is “temperature compensated,” i.e. that the period is almost the same at different constant ambient temperatures (Q_10_~1.0). These three properties define circadian rhythms, not any particular biochemical mechanism. The fascination of this phenomenon is to explain how a biochemical oscillator could have evolved that has such a long time constant (~24 h) which is both precise and temperature compensated.

Biologists have known for decades that circadian systems are practically ubiquitous among eukaryotic organisms, but these rhythms were originally thought to be absent from prokaryotes [5]. Beginning about 25 years ago, however, this dogma was overturned as research on prokaryotic cyanobacteria persuasively demonstrated that nitrogen fixation and gene expression exhibited *bona fide* circadian characteristics [5–7]. Once the floodgates of “eukaryotic-centric thinking” were opened, a torrent of information about circadian timekeeping in cyanobacteria was released, including that gene expression and chromosomal topology is globally regulated [8–13] in addition to circadian rhythms of nitrogen fixation, metabolism, and cell division [5–7,14–17]. Moreover, cyanobacteria have become the model system that most persuasively illustrates the adaptive value of circadian organization in 24-h rhythmic environments [2–4]. Three essential core clock genes have been identified in the cyanobacterium *Synechococcus elongatus* (*kaiA*, *kaiB*, and *kaiC* [18]), and the proteins they encode can reconstitute a circadian rhythm *in vitro* [19]. Salient properties of the central KaiABC pacemaker are a sustained rhythm of KaiC phosphorylation, a temperature-compensated ATPase activity of KaiC, and rhythmic interactions among the KaiA/KaiB/KaiC proteins [19–23].

Despite the findings in cyanobacteria, there are essentially no other prokaryotes for which *bona fide* circadian properties have been documented. Homologs of the essential *kaiB* and *kaiC* genes are distributed widely among eubacterial and Archaeal species [24,25], but a third essential gene–*kaiA*–is found only in the cyanobacteria. Perhaps a KaiB/KaiC “hourglass timer” is the more common timekeeping mechanism among prokaryotes, and the cyanobacterial KaiA/KaiB/KaiC circadian trio is an exceptional case? Even among the cyanobacteria, the *kaiA* gene is missing or truncated in the important cyanobacterial genus *Prochlorococcus*, which apparently has daily rhythms but not a sustained circadian rhythmicity in constant conditions [26,27]. One group has speculated that the cyanobacterial circadian clock has "de-evolved" to an hourglass timer in *kaiA*-free *Prochlorococcus* [28]. Despite the lack of documented circadian phenomenon among non-cyanobacterial prokaryotes, bacteria that live in daily light/dark cycles–especially those performing photosynthesis–would also be expected to benefit from a timing mechanism to adapt to these daily changes. Therefore, it is reasonable to predict that there are other bacterial species that possess a daily timekeeper, whether it be a circadian clock or an hourglass timer.

The purple non-sulfur *Rhodopseudomonas palustris* is a gram-negative bacterium that is unrelated to cyanobacteria (*R*. *palustris* is an alpha-proteobacterium) but harbors homologs of *kaiB* and *kaiC* (herein, *kaiB*^*Rp*^ & *kaiC*^*Rp*^). *R*. *palustris* is a metabolic "acrobat," as it can vault between four different modes of metabolism: photo-heterotrophic, photo-autotrophic, chemo-heterotrophic, and chemo-autotrophic [29]. Therefore it displays considerable metabolic versatility as it can grow aerobically or anaerobically, heterotropically or autotropically. Under anaerobic or microaerobic conditions in which *R*. *palustris* uses light to photosynthesize, it can also fix nitrogen. When it is in its photoautotrophic or photoheterotrophic modes, *R*. *palustris* relies upon sunlight for energy and therefore optimal adaptation to the daily light/dark cycle could involve the evolution of a daily timekeeper (as in the case of cyanobacteria), potentially to anticipate dawn and dusk so as to maximize the regulation of photosynthetic capacity. We therefore tested the possibility that *R*. *palustris* exhibits daily timekeeping properties that may or may not be circadian. We found that a *kaiC*-dependent system allows *R*. *palustris* to adapt optimally to daily light/dark cycles under photoheterotrophic conditions, but the characteristics of the daily patterns observed are not the same as those found in cyanobacteria. These data suggest that the timekeeping mechanism in *R*. *palustris* constitutes a “proto-circadian” system. By "proto-circadian," we mean a phenomenon that shows some–but perhaps not all–canonical properties of a *bona fide* circadian rhythm. This could be because the phenomenon is an evolutionary precursor under selection on a trajectory towards a complete circadian system, or because the ecological niche of the organism does not provide a selective advantage for some of the defining circadian properties and therefore they have not arisen in the organism's daily timekeeper. In the case of the *R*. *palustris* KaiB/C system, we suggest that it is an evolutionary precursor to the more robust and resilient KaiA/B/C system found in cyanobacteria.

## Results

Purple non-sulfur bacteria such as *R*. *palustris* are globally distributed in soil and water environments and they frequently cohabit niches with cyanobacteria [29,30,31,32], with the proviso that *R*. *palustris* prospers in multiple metabolic modes [29] whereas cyanobacteria prefer aerobic conditions. Therefore, the daily environmental conditions experienced by purple non-sulfur bacteria and cyanobacteria are similar, which might lead to similar selective pressures to evolve a daily timekeeping mechanism. As mentioned above, *R*. *palustris* also shares homologs of the clock genes *kaiB* and *kaiC* with cyanobacteria, although the order of *kaiB*^*Rp*^ and *kaiC*^*Rp*^ is reversed as compared with the *S*. *elongatus kaiABC*^*Se*^ trio, as shown in S1A Fig (however, the order is the same as for the *Synechocystis kaiCB* locus 3, S2 Fig). Moreover, the motif structure of *kaiC*^*Rp*^ is practically the same as for *kaiC*^Se^; both have a double domain CI/CII sequence with Walker A/B motifs, DXXG motifs, and catalytic EE residues in essentially the same locations (S1B Fig). The major differences between *kaiC*^*Rp*^ vs. *kaiC*^Se^ are that (1) the demonstrated phosphorylation sites in *kaiC*^Se^ are TST, whereas those same residues in *kaiC*^*Rp*^ are TSS, and (2) the C-terminal region in *kaiC*^*Rp*^ is longer, which may relate to the fact that this is the region of KaiC^Se^ that KaiA binds to stimulate KaiC^Se^ autophosphorylation [21,22]–since there is no KaiA in *R*. *palustris*, it is logical that this region may differ between *kaiC*^*Rp*^ and *kaiC*^Se^.

### Temporal patterns of nitrogen fixation in *R*. *palustris*

**Fig 1:**
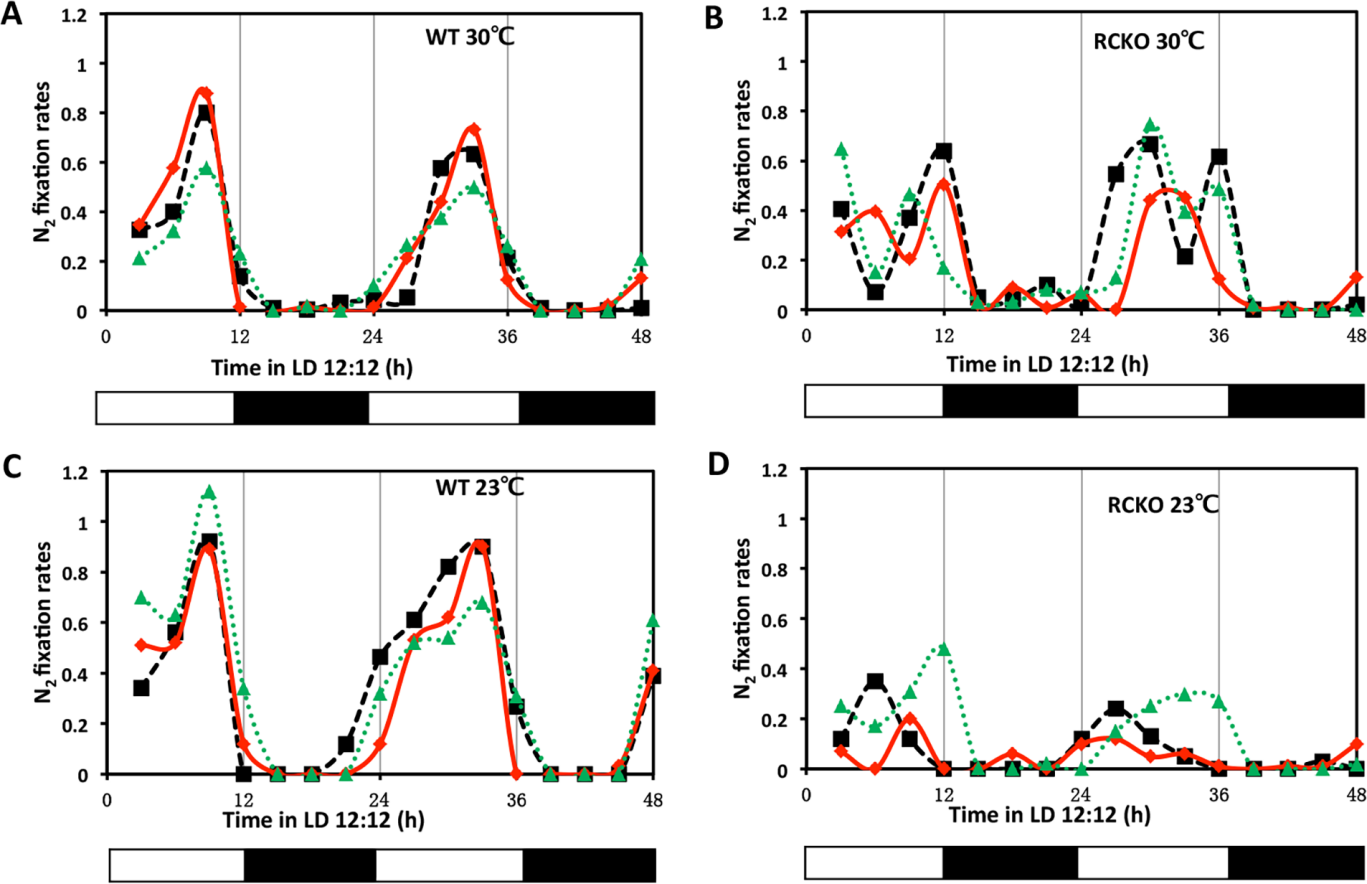
Daily patterns of nitrogen fixation in WT vs. RCKO strains. **A** and **C**, nitrogen fixation activities of the wild-type *R*. *palustris* (WT) at 30°C and 23°C. **B** and **D**, nitrogen fixation activities of the *kaiC*^*Rp*^-deletion strain (RCKO) at 30°C. The three traces represent three individual cultures under anaerobic conditions that were tested at different phases of LD 12:12 after growth for ~2 weeks in LD 12:12. The black and white bars underneath represent the light conditions. Nitrogen fixation rates were calculated based on the amount of C_2_H_4_ (nmol) produced by 10^10^ cells per hour. These two-cycle LD experiments were repeated twice, each time with 3 replicate cultures; one representative experiment is shown in this figure (one-cycle LD assays were conducted in five independent experiments, each time with phasing data equivalent to those shown in this figure). The data of Fig 1 are replotted in S3 Fig with all three replicate cultures averaged together (complete time series data appear in S4 Table).

**Fig 2:**
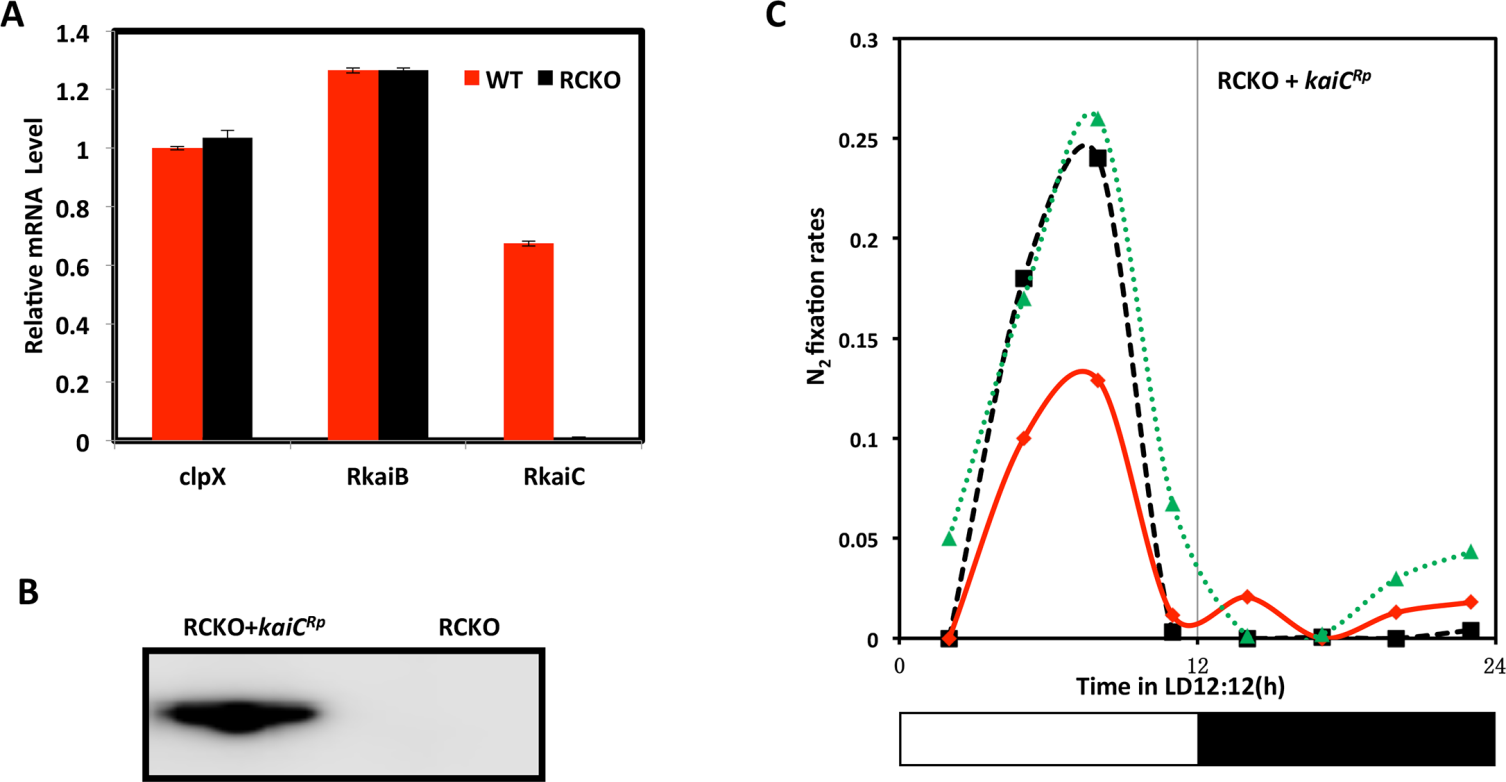
Characterizations of the RCKO and RCKO+*kaiC*^*Rp*^ strains. **A.** The mRNA levels of *kaiB*^*Rp*^ and *kaiC*^*Rp*^ were quantified by quantitative PCR from the WT (red) and RCKO (black) strains. The mRNA levels of *clpX* were included as an internal control. **B.** immunoblot by anti-FLAG antibody to confirm the expression of FLAG-KaiC^Rp^ in the rescued strain (RCKO+*kaiC*^*Rp*^). **C**. nitrogen fixation of the RCKO+*kaiC*^*Rp*^ strain under LD12:12 cycles at 30°C. Data using the HA-KaiC^Rp^ rescued strain are equivalent. The three traces represent three individual cultures. These experiments were repeated twice, each time with 3 replicate cultures; one representative experiment is shown in this figure (complete time series data for panel C appear in S5 Table).

These ecological and genetic similarities with cyanobacteria encouraged us to investigate whether *R*. *palustris* cells exhibit circadian properties. Under phototrophic conditions, the energy metabolism of *R*. *palustris* is at least partially dependent upon photosynthesis and therefore we expected that a circadian oscillator that allows the anticipation of the daily light cycle would be most likely to be expressed under phototrophic conditions. Inspired by the pioneers who first discovered circadian rhythms in cyanobacteria by monitoring nitrogen fixation [5,6,33], we measured the nitrogen fixation rate of *R*. *palustris* (strain TIE-1 [13]) under photoheterotrophic conditions in daily light/dark cycles (LD 12:12 = 12 h light/12 h dark). In *R*. *palustris*, nitrogen fixation is light-dependent and inhibited by oxygen. As shown in Fig 1A and 1C, under anaerobic conditions, nitrogen fixation peaked in the middle of the day in wild-type (WT) *R*. *palustris* at 23°C and 30°C. In WT, replicate cultures reproducibly peaked at the same phase in LD at both 23°C and 30°C, indicating at least some degree of entrainment and temperature compensation (the data of Fig 1 are replotted in S3 Fig with all three replicate cultures averaged together). We compared this daily pattern in WT with that of a strain in which the *kaiC*^*Rp*^ gene was knocked out (the RCKO strain) and of a *kaiC*^*Rp*^-knockout strain in which the *kaiC*^*Rp*^ gene was replaced at an ectopic site (the RCKO+*kaiC*^*Rp*^ strain). In RCKO, there is no expression of *kaiC*^*Rp*^ transcripts, but the level of *kaiB*^*Rp*^ transcript is equivalent to that of WT (Fig 2A), and the daily profile of nitrogen fixation is altered (Fig 1B and 1D). The nitrogen fixation of RCKO peaked in the day phase, but the particular phase adopted by three replicate cultures is highly variable (Fig 1B and 1D), unlike the stable phase relationship for WT replicates (Fig 1A and 1C, also see S3 Fig). In the RCKO+*kaiC*^*Rp*^ strain, expression of KaiC^Rp^ was rescued (Fig 2B) as well as a consistently phased nitrogen fixation pattern in LD12:12 (Fig 2C).

**Fig 3:**
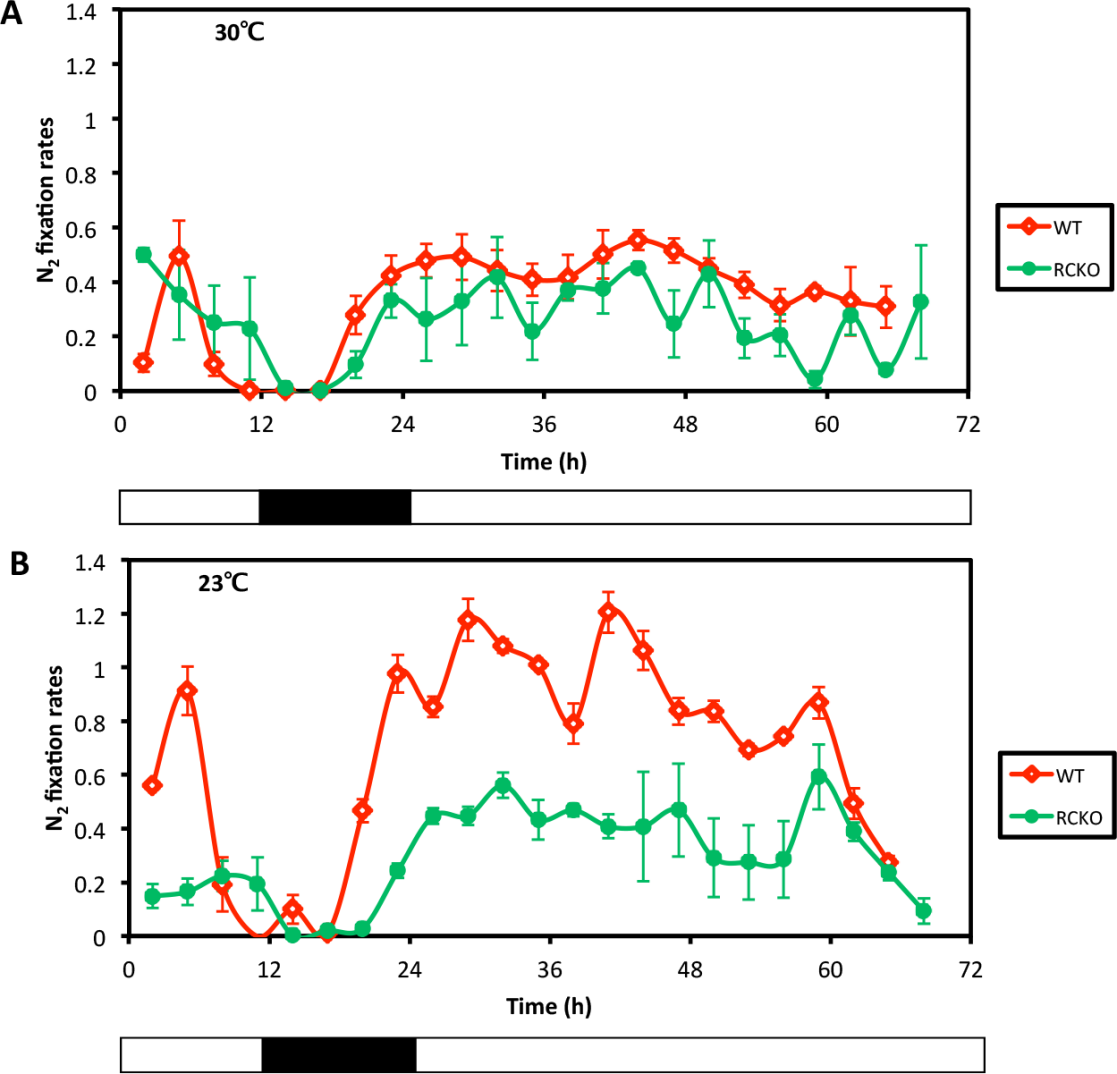
Lack of robust persistence of the nitrogen fixation rhythm in LL after transfer from LD. **A**, nitrogen fixation activities of the WT (red) and RCKO (green) strains at 30°C. **B**, nitrogen fixation activities of the WT (red) and RCKO (green) strains at 23°C. Data is plotted as the mean of three individual cultures. Data points are mean +/- S.D. Black-white bars indicate the light/dark conditions: black is dark, white is light. These experiments were repeated five times (five times for WT, and five times for RCKO), each time with three replicate cultures; one representative experiment is shown in this figure (the three replicate cultures of this experiment are plotted separately in S4 Fig, and complete time series data appear in S6 Table).

As explained above, a defining characteristic of *bona fide* circadian rhythms is their persistence in constant conditions, and the nitrogen fixation rhythm of cyanobacteria exhibits a robust rhythm in constant light (LL [5,6,33]). However, *R*. *palustris* did not exhibit a sustained rhythm when transferred from LD to LL. As shown in Fig 3, WT *R*. *palustris* cultures exhibited a robust rhythm in LD, but upon transfer to LL, high-amplitude rhythmicity was not sustained at 23°C or 30°C. Fig 3 illustrates the averaged results of multiple cultures, but the same effect is clear when individual cultures are plotted separately (S4 Fig). There was some suggestion of a low-amplitude rhythmicity in LL at both 23°C and 30°C with a free-running period (FRP) of approximately 22 h, but Cosinor analyses indicated that these periodicities were not significant (R^2^ is only 0.20 at 30°C, and a clearly non-significant 0.03 at 23°C; S5 Fig). Therefore, the daily rhythm of nitrogen fixation in WT *R*. *palustris* was clear in LD, but was either non-existent or highly damped in constant LL conditions, and therefore did not conform to the first defining property of circadian rhythmicity, namely robust persistence in constant conditions. In the RCKO strain, nitrogen fixation was not rhythmic by eye (Fig 3 and S4 Fig) or by Cosinor analysis (S5 Fig).

### Molecular correlates: KaiC^Rp^ ATPase activity and phosphorylation

**Fig 4:**
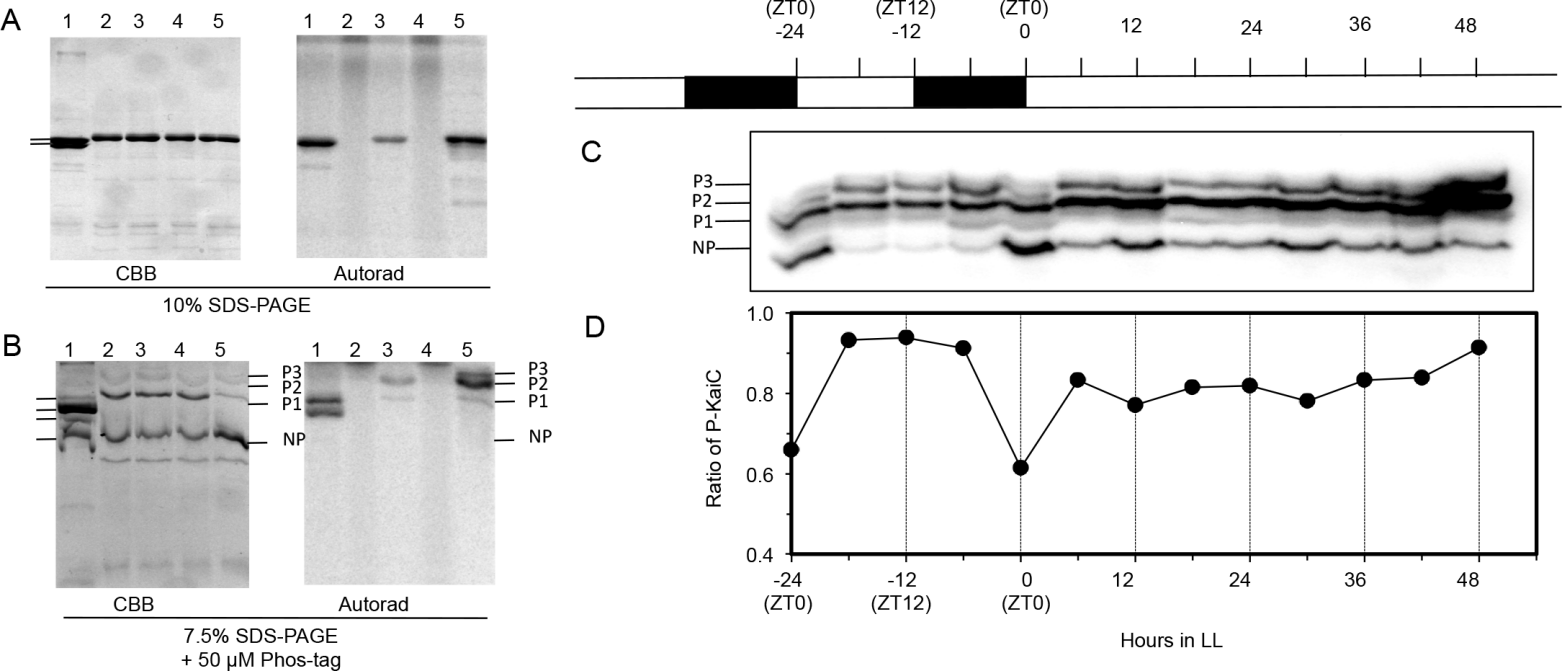
Phosphorylation patterns of KaiC^Rp^ in LD and LL. **A & B**. Electrophoresis analyses of ^32^P-labeled KaiC^Se^ and KaiC^Rp^. Lane 1 is ^32^P-labeled KaiC^Se^. Lanes 2–5 are purified KaiC^Rp^ that was mixed with [γ-^32^P]ATP. Samples for lanes 2 and 4 were immediately denatured after the addition of [γ-^32^P]ATP by mixing with SDS-PAGE sample buffer (time zero samples, lanes 2 and 4). Samples for lanes 3 and 5 were incubated with [γ-^32^P]ATP for 24 h at either 4°C (lane 3) or 30°C (lane 5) prior to SDS denaturation and inactivation. The samples were subjected to either regular SDS-PAGE (**A)** or phosphate-affinity SDS-PAGE with Phos-Tag (**B**). In both panels, the left portion is a Coomassie-Blue stained gel (CBB) and the right portion is the autoradiogram of P-32 radioactivity. In panel **B**, three bands of ^32^P-labeled KaiC^Rp^ are indicated as P1, P2, P3 and an unlabeled KaiC^Rp^ band is indicated as NP (non-phosphorylated). **C & D**. The strain in which KaiC^Rp^ has been tagged with HA (RCKO + HA-kaiC^Rp^) was grown under LD 12:12 and then transferred to LL. Cells were collected every 6 h. Total protein extracts were separated on SDS-polyacrylamide gels with Phos-tag and analyzed by immunoblotting using an anti-HA antibody. The specificity of the anti-HA antibody against HA-tagged KaiC^Rp^ was confirmed with extracts from the RCKO cells. **C.** immunoblot in which the phospho- (P1, P2, P3) and nonphospho- (NP) forms of KaiC^Rp^ are indicated. **D.** quantification of phosphorylation states of HA-KaiC^Rp^ from the immunoblot in panel C. “ZT” = Zeitgeber Time in LD, where ZT0 is lights-on and ZT12 is lights-off (complete time series data for panel D appear in S7 Table).

In the cyanobacterium *S*. *elongatus*, KaiC^Se^ undergoes robust rhythms in LL and LD of phosphorylation [21,34–36] and often rhythms of abundance as well [37]. Using the RCKO+*kaiC*^*R*^ strain (Fig 2), we assessed KaiC^Rp^ phosphorylation and abundance *in vivo* using an anti-HA antibody to the HA-tagged KaiC^Rp^ (Fig 4) after electrophoretic separation by Phos-Tag PAGE into different phosphoforms of KaiC^Rp^ (Fig 4). We generated different phosphoforms of KaiC^Rp^ by incubating the protein with P-32 labeled ATP overnight at 4°C and at 30°C (Fig 4A and 4B, lanes 2–5). Although SDS-PAGE separates the different phosphoforms of KaiC^Se^ [19,20,23,34,36](Fig 4A, lane 1), it does not separate the different phosphoforms of KaiC^Rp^ (Fig 4A, lanes 2–5). However, SDS-PAGE including Phos-Tag reagent can separate phosphorylated KaiC^Rp^ into at least three differentially migrating variants, labeled P1, P2, and P3 in Fig 4B (non-phosphorylated KaiC^Rp^ is labeled NP). When the Phos-Tag PAGE is used to analyze extracts from *R*. *palustris* cells in LD and LL, we found a clear daily rhythm of KaiC^Rp^ phosphorylation *in vivo* in LD, with a trough of KaiC^Rp^ phosphorylation at the end of the night phase. However, this daily rhythm either disappears or is highly damped in LL, which is the same pattern seen with nitrogen fixation (Fig 3). Moreover, there is not an obvious 24-h cycle of KaiC abundance in LL. Therefore, KaiC^Rp^ exhibits a daily–but not a circadian–rhythm of phosphorylation status *in vivo*.

**Fig 5:**
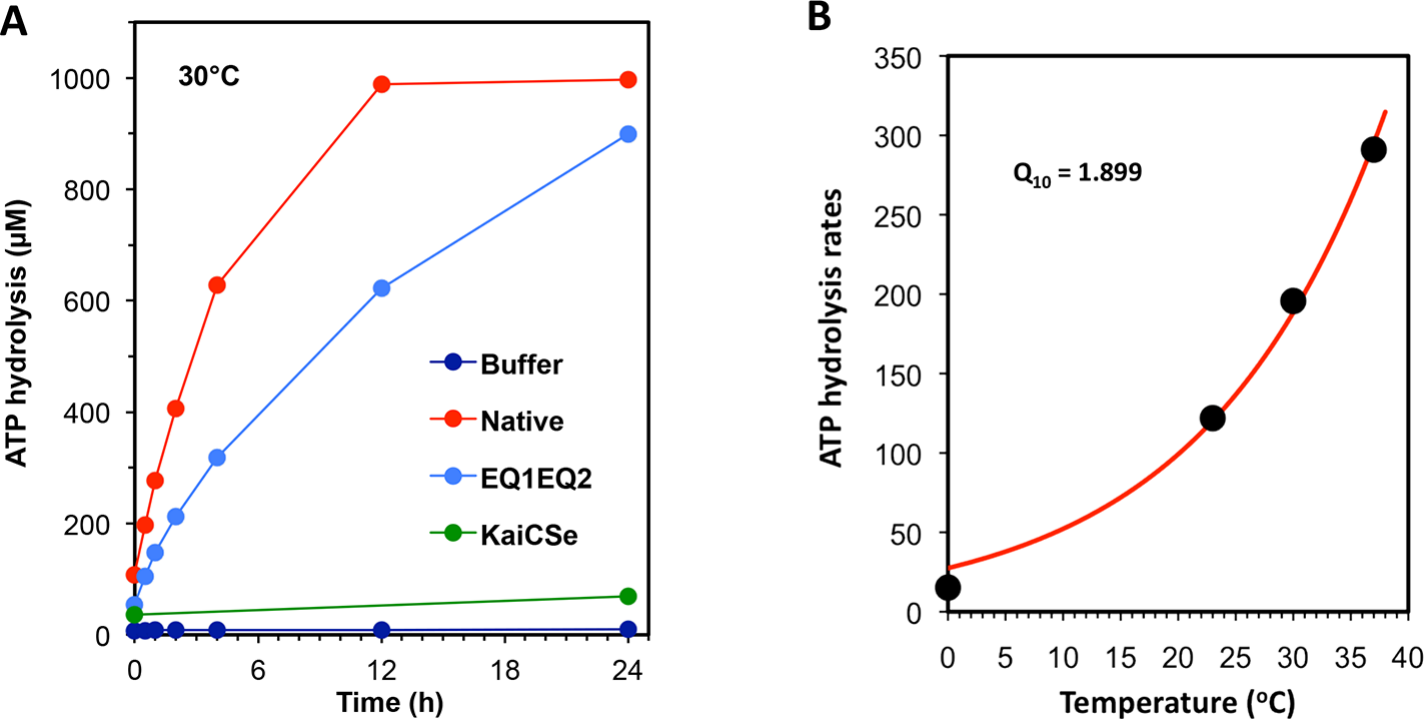
ATPase activity of KaiC^Rp^ and KaiC^Rp-EQ1EQ2^ *in vitro* at 30°C. **A.** Purified native KaiC^Rp^ and the KaiC^Rp-EQ1EQ2^ mutant (EQ1EQ2) were incubated with1 mM ATP for 0.5, 1, 2, 4, 12, and 24 h at 30°C. The amount of ATP hydrolyzed was quantified by HPLC. Dark blue, buffer only; red, KaiC^Rp^; light blue, KaiC^Rp-EQ1EQ2^; green, KaiC^Se^. **B.** ATPase hydrolysis rates of native KaiC^Rp^ at different temperatures. Q_10_ was calculated based on these rates. These experiments were repeated twice; one representative experiment is shown in this figure.

Another key property of KaiC^Se^ is its ability to hydrolyze ATP by a reaction that is very slow (15 ATP molecules hydrolyzed per day per KaiC^Se^ monomer at 30°C) and temperature-compensated (Q_10_ ~ 1.0 [38,39]). This ATPase reaction has been proposed to be the core biochemical reaction underlying circadian periodicity in cyanobacteria [38]. The ATPase activity of KaiC^Se^ is significantly reduced when the four catalytic glutamate (E) residues (S1B Fig) are replaced with glutamine (Q) residues [39]. While KaiC^Rp^ hydrolyzes ATP *in vitro* and this activity is decreased when the four catalytic EE residues are replaced with glutamine residues (EQ1EQ2), the rate of ATP hydrolysis by native KaiC^Rp^ was >100X higher than that for KaiC^Se^ at 30°C (Fig 5A). The ATPase activity of KaiC^Rp^ co-migrates with the abundance profile of the purified protein upon gel filtration chromatography, indicating that the measured activities are those of the KaiC proteins and not of a putative contaminant (S6 Fig). Unlike the case for KaiC^Se^, the rate of ATP hydrolysis by KaiC^Rp^ was not temperature compensated (Q_10_ = 1.9, Fig 5B).

### KaiC-dependent growth in LD versus LL

**Fig 6:**
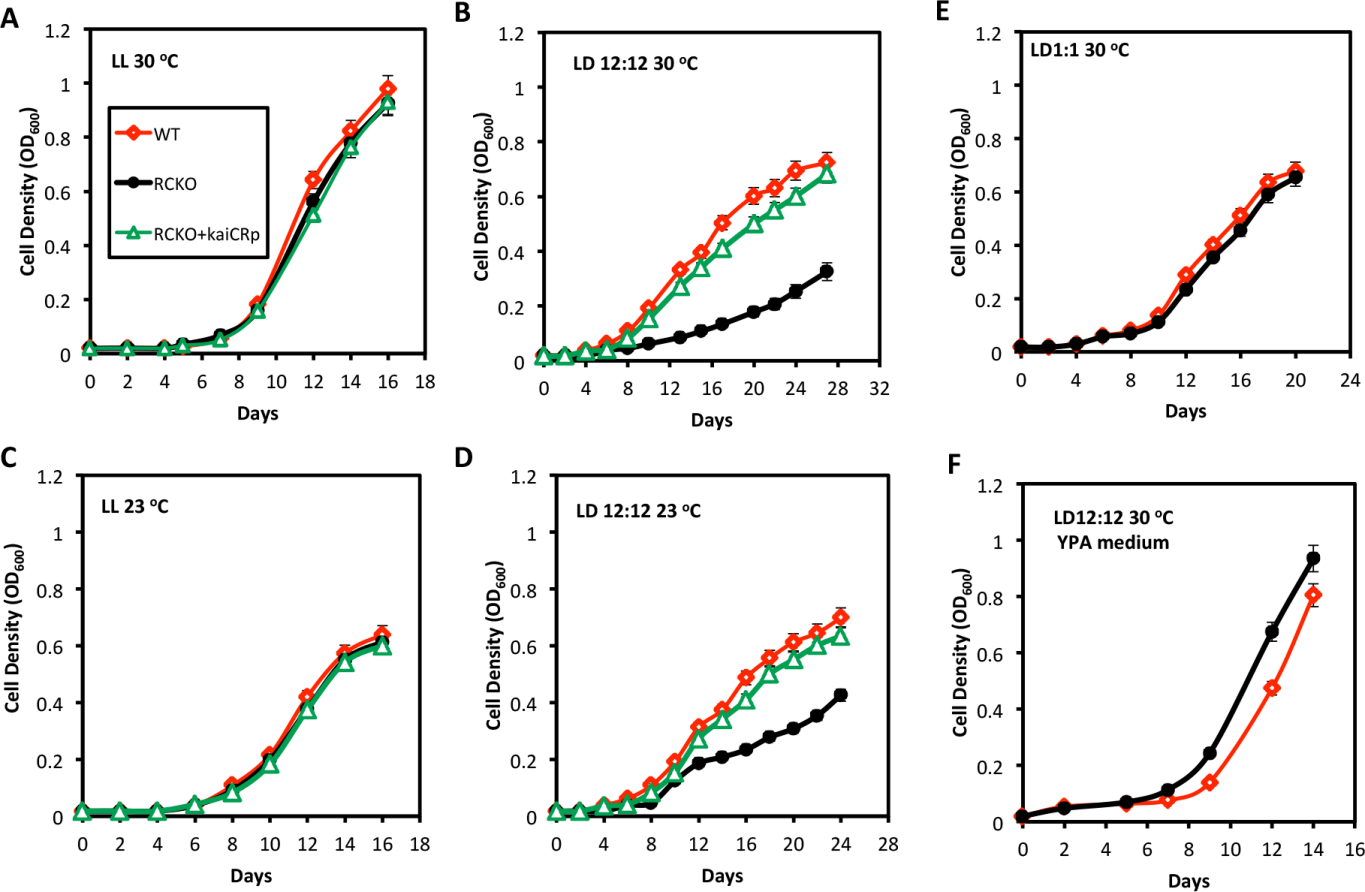
Growth rate of *R*. *palustris* is *kaiC*^*Rp*^-dependent in cyclic 24-h conditions. Cell densities (OD_600_) of the WT (red), RCKO (black) and RCKO-*kaiC*^*Rp*^ (green) strains were measured every two days under (**A**) LL conditions at 30°C; (**B**) LD 12:12 cycles at 30°C; (**C**) LL conditions at 23°C; (**D**) LD 12:12 cycles at 23°C; (**E**) LD 1:1 cycles at 30°C; (**F**) LD 12:12 cycles at 30°C in YPA medium. All cultures were grown anaerobically. These experiments were repeated three times, and each time there were three replicate cultures. In this figure, the data from the three separate experiments were pooled and plotted as the mean +/- S.D. (therefore, each datum is the average of n = 9).

Despite the lack of a robust free-running rhythm in LL (Fig 3), KaiC^Rp^ clearly affected the timing of temporal events in LD (Fig 1, S3 Fig). We reasoned that consistent temporal patterning in LD (the typical environmental condition) might enhance fitness even if the rhythmicity does not persist robustly in LL (which is, after all, a special or non-environmental condition). For microbial organisms, growth rates are an excellent gauge of fitness. The growth rates of all three strains (WT, RCKO, and RCKO+*kaiC*^*Rp*^) were equivalent in the non-rhythmic LL condition under which the cells grew photoheterotrophically (Fig 6A and 6C). Therefore, knocking out the *kaiC*^*Rp*^ gene had no detectable effect upon the growth of cells under noncyclic photoheterotrophic conditions.

However, the result was completely different under temporally cycling photoheterotrophic conditions. In a light/dark cycle of 12 h light, 12 h darkness (LD 12:12), the RCKO strain grew significantly slower at either 23°C or 30°C (Fig 6B and 6D). This diminution of growth rate was clearly *kaiC*^*Rp*^–dependent because the replacement of *kaiC*^*Rp*^ to the RCKO strain rescued the growth defect in LD 12:12 (RCKO-*kaiC*^*Rp*^ strain, Fig 6B and 6D), but had no detectable impact under LL conditions (Fig 6A and 6C). Under chemoheterotrophic growth conditions in YPA medium, however, WT and RCKO grew at the same rate in LD (Fig 6F). Consequently, knocking out the *kaiC*^*Rp*^ gene did not affect growth under chemoheterotrophic conditions in LD.

It might be argued that the *kaiC*^*Rp*^–dependent growth effect under photoheterotrophic conditions in LD may be due to the reduced total amount of light in LD vs. LL, because LD 12:12 has half the amount of light as LL over time. We therefore tested LD 1:1 (1 h light/1h dark) cycles that also have half the amount of light as LL, but in which the cycle has no 24-h information. Under this condition, there was no difference in the growth rate between WT and RCKO (Fig 6E). Consequently, functionally KaiC^Rp^ had an important effect on photoheterotrophic growth/fitness in 24-h cyclic environments (LD 12:12), but not in environments without 24-h cycles (LL or LD 1:1) or under chemoheterotrophic conditions when energy metabolism is not dependent upon light (Fig 6).

## Discussion

### A proto-circadian system in *Rhodopseudomonas* that is adaptive

Numerous bioinformatic studies have noted the wide distribution of *kaiB* and *kaiC* homologs among many prokaryotic groups, including Archaea [24,25]. Based upon the discovery of circadian rhythms in *S*. *elongatus* and its essential *kaiABC* clock gene cluster [7,18], these bioinformatic studies speculated that many prokaryotic species might harbor circadian systems [24,25]. Nevertheless, attempts to discover *bona fide* circadian rhythms in prokaryotes outside the cyanobacteria have been frustratingly unsuccessful [5]. An example is a study of “rhythmic” gene expression in the purple bacterium *Rhodobacter sphaeroides*, where noisy circa-20 h oscillations of a reporter were observed under aerobic conditions, but neither temperature compensation nor entrainment was reported [40]. Therefore, even though that genome includes *a kaiC-B-PAS/HK* operon (as in *R*. *palustris*, S1B Fig), it is impossible to conclude whether *Rhodobacter* has a circadian system or not. In many other prokaryotic species that have been tested, observing consistent persisting circa-24-h rhythms has been elusive, let alone temperature compensation or a rigorous demonstration of entrainment [5].

As reported herein, *R*. *palustris* exhibits some *kaiC*-dependent properties that are consistent with a circadian-type system, including a phase angle in LD that is consistent at different temperatures (implying temperature compensation), and slowing and suppressing of circadian rhythms in cyanobacteria when KaiC^Rp^ is expressed in the heterologous *S*. *elongatus* (S7 Fig). Nevertheless, other observations from *R*. *palustris* are unlike those found in *S*. *elongatus*, such as (1) the nitrogen fixation rhythm in constant conditions is either not consistently rhythmic or is highly damped (not well sustained), (2) there is no 24-h rhythm of KaiC^Rp^ phosphorylation status or abundance in constant LL conditions, and (3) the ATPase activity of KaiC^Rp^ is significantly higher than that of KaiC^Se^ and not temperature compensated. It might be argued that the nitrogen fixation rhythm is not well coupled to a putative central pacemaker in *R*. *palustris* (unlike the case in cyanobacteria [5,6,33]) and a different parameter would be a better indicator, but this suggestion misses the point that monitoring one output rhythm after another [5] may be a fruitless endeavor and that the standards established for circadian-type systems in eukaryotes may not be the optimal criteria when studying prokaryotes.

We have taken a different approach with *R*. *palustris* that focused upon criteria related to adaptive significance rather than canonical properties. The circadian system in *S*. *elongatus* has been rigorously established to provide a fitness advantage in 24-h rhythmic environments, but not in constant non-selective environments [2–4]. We found that growth of *R*. *palustris* under photoheterotrophic conditions is clearly dependent upon *kaiC* in 24-h cyclic environments, but not in constant (non-24-h cycles) environments (Fig 6). Therefore, despite the lack of a robustly sustained nitrogen fixation rhythm (Fig 3, S4 Fig) or rhythms of KaiC phosphorylation (Fig 4) in constant conditions, *R*. *palustris* has a *kaiC*-dependent timekeeping system that has at least some circadian-like properties. Moreover, the anticipation of dawn by the nitrogen fixation pattern in LD (i.e., the fact that nitrogen fixation activity is rising at the end of the night before lights-on) that is obvious in Fig 1C and Fig 3A and 3B implies a timekeeper rather than a direct light/dark response. It is tempting to conclude that the daily *phasing* of metabolic events (exemplified by nitrogen fixation, Fig 1) among the WT, RCKO, and RCKO+*kaiC*^*Rp*^ strains is responsible for the differential growth rates (Fig 6). However, there may be other *kaiC*^Rp^-dependent factors that are responsible for the differential growth rates–but if so, these factors must be dependent upon an interaction between KaiC^Rp^ and a rhythmic environment with a 24-h periodicity. A fully operational circadian system may underlie the phenomena we observed in *R*. *palustris*. However, the lack of a robustly persisting rhythm in constant conditions coupled with properties of KaiC^Rp^ that clearly differ from those of KaiC^Se^ persuade us to be more cautious. We propose instead that the timekeeping system in *R*. *palustris* is more likely to be a “proto-circadian oscillator” in which natural selection has not yet selected for a sufficiently resilient clock that it sustains itself in constant conditions.

### Evolution of clocks and new criteria for circadian-type timekeepers in prokaryotes

If the proto-clock in *R*. *palustris* is adaptive despite the absence of a persisting free-running rhythm, why has evolution repeatedly selected for circadian rhythms in eukaryotes that persist robustly and sustain free-running rhythms in constant conditions? There is presently no clear answer why biological oscillators that persist in constant conditions (a condition that is rare in nature–restricted to caves or to polar regions in summer or winter) were selected over hourglass timers to be the biological timekeepers for organisms in natural light/dark (LD) cycles. Roenneberg and Merrow have stated, “Evolution has shaped circadian clocks in a cyclic world; temporal constancy of environmental qualities must have been an extremely rare exception. It is therefore the mechanism of entrainment that has evolved and not sustained rhythmicity in constant conditions. The latter is theoretically not even essential for a functional entrained circadian system” [41]. The case of *R*. *palustris* is an excellent example that supports the foregoing statement. What might be the selective forces that would encourage a self-sustained circadian clock? Some modelers have suggested that environmental noise (e.g., light and temperature fluctuations) strongly select for the complexity of a circadian timekeeper that sustains itself after being transferred to unnatural constant conditions [42]. The environmental noise used in these simulations included daily fluctuations in light intensity & temperature as well as annual changes in photoperiod (however, organisms that live near the equator and experience practically no annual cycle of photoperiod nevertheless evolved self-sustained circadian oscillators).

The comparison among *Rhodopseudomonas*, *Synechococcus*, and *Prochlorococcus* might be enlightening in this regard. Cyanobacteria such as *S*. *elongatus* are the only prokaryotic group to have the *kaiA* gene. The known function of the KaiA protein in cyanobacteria is to bind to the C-terminal tentacles of KaiC and stimulate the phosphorylation of KaiC^Se^ [21,22,34,36]. Consistent with the absence of KaiA from *R*. *palustris*, the KaiA-binding C-terminal tentacles of KaiC^Se^ are significantly different from the corresponding regions of KaiC^Rp^ (an obvious difference from S1B Fig is that the C-terminal regions of KaiC^Rp^ are longer than those of KaiC^Se^, but the sequence of residues is also different). In addition, KaiA is sequestered during the KaiC^Se^ dephosphorylation phase, thereby inactivating its activity; this feature of KaiA regulation assists the synchronization and robustness of the cyanobacterial KaiABC oscillation [21,22,36]. Perhaps the inclusion of KaiA is an evolutionary innovation in cyanobacteria that enhances the sustainment of *kaiB/kaiC*-based oscillations, resulting in the exquisite rhythms of cyanobacteria in LL [7,21,22]. We can infer this conclusion both because the *kaiB/kaiC*-based system without *kaiA* of *R*. *palustris* is not well sustained (this report), but also because the widespread marine cyanobacterium *Prochlorococcus* “lost” the *kaiA* gene in its evolution (non-functional vestiges of *kaiA* remain in the *Prochlorococcus* genome [24,25,28]; *Prochlorococcus* also does not sustain robust rhythms in constant conditions [26–28]. Given that the complete *kaiABC* system is adaptive in the soil/freshwater cyanobacterium *S*. *elongatus* [2–4], why might selection have relaxed for maintaining *kaiA* in *Prochlorococcus*? The explanation may lie in the relatively consistent marine environment of *Prochlorococcus*. As suggested by other researchers [26–28], in the interests of genomic streamlining *Prochlorococcus* could afford to lose the sustained *kaiABC* system in favor of a reduced *kaiBC* system because the daily rhythmicity of its marine environment is less noisy than that of most terrestrial/aquatic environments.

Whether those evolutionary speculations be true or not, our study establishes a new set of criteria for circadian-type timekeeping systems in prokaryotes that harbor *kaiC* and/or *kaiB* homologs based on adaptiveness rather than upon expression of rhythmic parametrics. In particular, the experiments depicted in Fig 6 demonstrate *kai*-dependent growth under selective (24-h cycles) versus non-selective conditions. This approach can be applied to many prokaryotic species. A particularly interesting example would be the application of this criterion to prokaryotic species that harbor only *kaiC* homologs to address the question of whether the ancestral role of *kaiC* (a RecA/DnaB-related gene [43]) has no rhythmicity function, and the evolutionary addition of *kaiB* conferred the adaptive timekeeping properties observed in *R*. *palustris* and *Prochlorococcus*.

## Materials and Methods

### Bacterial strains and culture conditions

All bacterial strains used in this study are listed in S1 Table. *E*.*coli* was grown in Luria-Bertani (LB) broth at 37°C with shaking, and when relevant, gentamicin (50 μg/ml) was added. For photoheterotrophic growth, *R*. *palustris* strains were grown in Freshwater-Base (FW) medium [30] supplied with 20 mM sodium acetate, 50 mM sodium bicarbonate, 20 mM MOPS (pH 7.2), 1 mM potassium phosphate, 1 mM sodium sulfate, multivitamin solution and trace elements solution. The medium was aliquoted into sealed serum bottles and the headspace was flushed with N_2_ gas for 20 min before inoculating cells. N_2_ gas was used as the sole nitrogen source to ensure nitrogenase expression. The cultures were maintained at 30°C (or 23°C as specified) and illuminated with cool-white fluorescent lamps (40–50 μE m^−2^s^−1^) with gentle shaking. For aerobic chemoheterotrophic growth, *R*. *palustris* strains were grown in YPA medium containing 0.3% yeast extract and 0.3% peptone (= YPA medium, [30]) at 30°C with gentle shaking, and when relevant, gentamicin (400 μg/ml) was added. Cyanobacteria (*S*. *elongatus* reporter strain AMC149 [7]) was grown in BG-11 medium [44] at 30°C, and illuminated by cool-white fluorescence lamps (40–50 μE m^−2^s^−1^) with air bubbling. The medium of AMC149 was supplemented with spectinomycin (25 μg/ml). For growth on solid media, 1.5% agar was included with the LB, FW or BG-11 medium and appropriate antibiotics.

### Construction of the *kaiC*^*Rp*^ deletion strain

All plasmids and primers used in constructing strains are listed in S2 Table and S3 Table. The *kaiC*^*Rp*^ deletion strain (RCKO) was constructed in *R*. *palustris* TIE-1 strain by overlap extension PCR and conjugation [45,46]. To delete the *kaiC*^*Rp*^ gene, the 1 kb upstream region and the 1 kb downstream region of the *kaiC*^*Rp*^ ORF were cloned from the genomic DNA of *R*. *palustris* TIE-1 strain and fused by overlap extension PCR. The 2-kb DNA fragment was ligated with the suicide vector pJQ-200KS [47]. The resulting plasmid, pJQ-200KS-RCKO, was transformed into *R*. *palustris* TIE-1 strain by conjugation with *E*. *coli* S17-1 [45,46,48]. The integration of the plasmid in either the upstream or the downstream region of the *kaiC*^*Rp*^ locus was selected by gentamicin resistance and screened by PCR. Following the selection, the integrants were grown in non-selective YPA medium for several generations and then plated on YPA agar medium with 10% sucrose [45] to induce double recombination. Among the survivors of the sucrose-YP medium, the double recombinants were selected by PCR screening. The deletion of the *kaiC*^*Rp*^ gene was confirmed by sequencing and Q-RT-PCR.

### Construction of the FLAG-*kaiC*^*Rp*^ strain and HA- *kaiC*^*Rp*^ strain

To complement the *kaiC*^*Rp*^ gene deletion, a FLAG-tagged or HA-tagged *kaiC*^*Rp*^ gene was restored to the genome of RCKO strain in the region surrounding the *glmUSX-recG* locus [45]. These tags allow us to confirm the expression of KaiC^Rp^ in the rescued strain with the appropriate anti-tag antibody. To construct the insertion plasmid pJQ200KS-insert, the 1 kb upstream and the 1 kb downstream regions of the *glmUSX-recG* locus of *R*. *palustris* TIE-1 were cloned and fused by overlap extension PCR. A *NcoI* site was incorporated in the middle to allow the insertion of genes of interest [45]. The resulting DNA fragment was ligated with pJQ200KS by *Sph*I and *Sma*I, as described by Bose and Newman [45,47]. To include the native promoter region of the *kaiC*^*Rp*^ gene in the insertion plasmid, a 469-bp region upstream of the *kaiC*^*Rp*^ gene was cloned and ligated to the *NcoI* site of pJQ-200KS-Insert, resulting in pJQ200KS-Insert-PkaiC^Rp^. A *NdeI-XbaI* site was incorporated downstream of the promoter region to allow the insertion of genes of interest. The *KaiC*^*Rp*^ gene was cloned from genomic DNA of the *R*. *palustris* TIE-1 strain, while a FLAG tag or HA tag was fused to its N-terminus. This DNA fragment was ligated with the plasmid pJQ200KS-Insert-PkaiC^Rp^ by *NdeI* and *XbaI* where the FLAG- *kaiC*^*Rp*^ or HA- *kaiC*^*Rp*^ gene is under the control of the *kaiC*^*Rp*^ promoter. The resultant plasmid, pJQ200KS-Insert-PkaiC^Rp^-FLAGkaiC^Rp^, or pJQ200KS-Insert-PkaiC^Rp^-HAkaiC^Rp^, was then transformed into RCKO strain by conjugation with *E*.*coli* S17-1 [45,46,48]. The selection procedures were similar to those used to construct the *kaiC*^*Rp*^ deletion strain. The integration of FLAG-*kaiC*^*Rp*^ and HA- *kaiC*^*Rp*^ was confirmed by sequencing and immunoblotting.

### Growth experiments

Batch liquid cultures of *R*. *palustris* strains including the wild-type *R*. *palustris* TIE-1, the *kaiC*^*Rp*^ knockout strain RCKO, and the *kaiC*^*Rp*^ complemented strain RCKO-FLAG*kaiC*^*Rp*^ were grown anaerobically in FW or YPA medium. For growth curve experiments and doubling time calculations, cultures were grown at 30°C or 23°C with gentle shaking under either LL conditions or LD (LD 12:12 or LD 1:1) conditions to test fitness by growth rate (as done for cyanobacteria in [49]). Seed cultures of these strains were grown anaerobically in FW medium under LL before inoculation. Growth was monitored by measuring the optical density (OD) at 600 nm.

### Nitrogenase activity measurement

Nitrogenase activity of *R*. *palustris* was measured by the acetylene reduction assay [50]. Assays were carried out in sealed serum bottles containing the anaerobic cultures under LL or LD conditions. At each time point, 3 mL of the culture was withdrawn anaerobically, injected into a 7-mL blood collection tube (Becton Dickinson Vacutainer, 8020128) containing 10% acetylene gas (final concentration) and 90% nitrogen gas (final concentration) and incubated under light (40–50 μE m^−2^s^−1^) for 3 h. The reaction was stopped by injecting 100 μL of 0.5 M EDTA and stored at room temperature until analyzed by gas chromatography. For time-course experiments, samples were taken every 3 hours. A 500 μl aliquot of the gas phase from the headspace was analyzed by a gas chromatograph (Shimadzu GC-2010 Plus) fitted with a flame ionization detector and a Rt-Alumina BOND/MAPD PLOT column (Restech, PA). The temperatures of the injector, detector, and oven were 200°C, 200°C, and 130°C, respectively.

### Phylogenetic analysis

The amino acid sequences of KaiC proteins from selected cyanobacterial species and purple non-sulfur bacterial species were retrieved from NCBI GenBank. The phylogenetic tree for KaiC was constructed using Phylogeny.fr web service integrated MUSCLE alignment, Gblocks curation and PhyML method [51–57]. The KaiC sequences were aligned and compared using PRALINE multiple sequence alignment program with the homology-extended alignment strategy [58–61].

### Quantitative reverse-transcription PCR

RNA was isolated from 3–5 ml of exponentially growing *R*. *palustris* cells with a NucleoSpin RNA II kit (Clontech). 300 ng of RNA was then used to synthesize cDNA by using the iScript cDNA synthesis kit (Bio-Rad). With 1 μl of the synthesized cDNA as template, quantitative reverse-transcription PRC was conducted on a CFX96 Touch Real-Time PCR Detection System (Bio-Rad) by using iTaq SYBR green supermix (Bio-Rad). The program was run at 95°C for 30 s, followed by 40 cycles of 95°C for 5 s and 60°C for 30 s. A final melting curve was performed for each reaction to ensure that only a single peak was amplified. The primers were designed by using Primer3 web service (http://biotools.umassmed.edu/bioapps/primer3_www.cgi) and shown in S3 Table.

### Phosphorylation assay for KaiC^Rp^

KaiC^Se^ was labeled with P-32 by incorporation of γ-phosphate from ^32^P-labeled ATP as previously described [62]. To label KaiC^Rp^, purified KaiC^Rp^ (0.2 μg/μL) was incubated at either 4°C or 30°C in 20 mM Tris-HCl, pH 8.0, 150 mM NaCl, 5 mM MgCl_2_, 1 mM DTT, 1 mM ATP and 0.22 μM [γ-^32^P]ATP (3000 Ci/mmol, PerkinElmer) for 24 hours. The phosphorylation reactions were terminated by mixing with SDS-PAGE sample buffer, heated at 96°C for 10 min, and subjected to either regular SDS-PAGE (10% acrylamide) or phosphate affinity SDS-PAGE (7.5% acrylamide with 50 μM Phos-Tag and 100 μM MnCl_2_)[63]. Gels were fixed, stained with colloidal Coomassie Brilliant Blue G-250, dried and imaged by a CCD camera (for CBB staining) or storage phosphor imaging technique (for ^32^P-autoradiography).

### Immunoblot assay for HA-KaiC^Rp^

The HA-*kaiC*^*Rp*^ strain was cultured in LD 12:12 cycles under anaerobic conditions. When the cell density (OD_600_) reached 0.4 in the exponential growth phase, 5 ml cells were collected every 6 h for 24 h in LD and then the culture was released to LL, under which conditions the cell collection was continued for another 48 h. After each collection, cells were centrifuged at 4°C, and cell pellets were immediately flash-frozen with liquid nitrogen. Total protein was extracted from cell pellets that had been resuspended in KaiC extraction buffer [37] by sonication, and the amount of total protein was quantified with the Lowry protein assay [64]. Equal amounts of total protein (10 μg) were placed into each well, and the proteins in the extracts were separated by phosphate affinity SDS-PAGE (7.5% acrylamide with 50 μM Phos-tag and 100 μM MnCl_2_) [63] and transferred onto polyvinylidene difluoride (PVDF) membranes. The immunoblots were treated with 0.5 μg/mL anti-HA monoclonal antibody (HA.11 clone 16B12, Covance, 1:2000 dilution) and detected with Pierce ECL Western Blotting Substrate (Thermo Scientific). The signal was captured by an Alpha Innotech gel image system (Cell Biosciences).

### Purification of KaiC proteins and ATPase activity assay

The *kaiC*^*Rp*^ gene was cloned from the genomic DNA of *R*. *palustris*. A GST tag was added into the N-terminus for protein purification. Using the QuickChange II kit (Agilent), site-directed mutagenesis was conducted to generate mutated KaiC^Rp^ in which glutamate (E) residues were replaced with glutamine (Q) residues. The KaiC^Rp-EQ1EQ2^ mutant had the four catalytic E residues (two in the CI domain, two in the CII domain) replaced by Q residues. GST-tagged KaiC^Rp^ and KaiC^Rp-EQ1EQ2^ was expressed and purified in *E*. *coli* DH5α cells following the protocol of expressing and purifying KaiC^Se^ [19,23]. GST fusion proteins were purified by affinity chromatography on glutathione-agarose resin (Pierce/Thermo Scientific) in buffer containing 20 mM Tris-HCl (pH 8.0), 300 mM NaCl, 5 mM MgCl_2_ and 1 mM ATP, and cleaved from GST using human rhinovirus 3C protease. The proteins were further purified by ion-exchange chromatography on Q Sepharose (GE Healthcare Life Sciences, 17-1014-01) with a gradient of NaCl. To confirm the purity of the KaiC^Rp^ proteins, some preparations were further subjected to gel filtration chromatography (Superdex 200 HR 10/30, GE Healthcare Life Sciences) to verify co-migration of the ATPase activity with KaiC^Rp^ (S6 Fig).

To assay ATPase activity, purified KaiC^Rp^ and KaiC^Rp-EQ1EQ2^ proteins were incubated in 20 mM Tris-HCl (pH 8.0), 165 mM NaCl, 5 mM MgCl_2_, 1 mM ATP, 0.5 mM EDTA at 0, 23, 30 and 37°C for 0.5, 1, 2, 4, 12 and 24 hours. At each time point, 15 μL of the reaction was withdrawn and mixed with 75 μL of 260 mM sodium phosphate, 10 mM EDTA, pH 5.0 (= 6-fold dilution), snap-frozen in liquid nitrogen and stored at -80°C until the day of measurement. The hydrolysis of ATP by the KaiC proteins were quantified using high-performance liquid chromatography (HPLC) as described [65] by using a Synergi Polar-RP column (250 × 4.6 mm, Phenomenex) with an isocratic mobile phase of 260 mM sodium phosphate, pH 5.0 at a flow rate of 0.8 mL/min. Ten μL of the sample was injected and the eluents were monitored at a wavelength of 259 nm. Peak areas of analytes were obtained from each chromatogram by the software and inspected manually. The linearity of the detector response was assessed by injecting a wide range of known concentrations (1.66–166 μM) of the nucleotide, and a standard curve was generated by plotting adenine nucleotide concentrations versus peak area. The analyte concentrations of the samples were calculated from the standard curve.

### Statistical analyses

Statistical analyses were performed in R. The modified cosinor method [66] was used to determine if the data were rhythmic or not on a 12-24-h time basis. The nitrogen fixation data were detrended linearly [65], and the detrended data were fitted to a series of cosine curves with different periods ranging from 12 h to 24 h. Along with curve fitting, the R^2^ value was calculated. Sample and replicate numbers are indicated in the legend to S5 Fig.

## Supporting Information

S1 Text Supplemental Methods. Including: Construction of *kaiC*^*Rp*^ expression strains in *S*. *elongatus*, Measuring luminescence rhythms in cyanobacterial strains.
(PDF)

S1 Fig Similar *kaiBC* genes of *R*. *palustris* and *S*. *elongatus*. **(A)** Arrangement of *kaiA*^*Se*^, *kaiB*^*Se*^, and *kaiC*^*Se*^ genes in the genome of *S*. *elongatus* (separate promoters drive expression of *kaiA* and *kaiBC* transcripts) as compared with *kaiC*^*Rp*^, *kaiB*^*Rp*^, and a histidine kinase gene with a PAS domain in *R*. *palustris* (bioinformatic analyses suggest that a single promoter drives expression of these three genes). **(B)** Comparison of motifs found in *kaiC*^*Rp*^ versus *kaiC*^*Se*^ shows the similarity of these genes. Both of the *kaiC* genes contain two RecA-like NTPase superfamily domains that include Walker A and Walker B motifs, catalytic EE residues, and DXXG motifs. The known phosphorylation sites of KaiC^Se^ are located in the second domain (CII) as TST, while in KaiC^Rp^ they are TSS. The C-terminus of KaiC^Rp^ is about 50 amino acid longer than that of KaiC^Se^.
(PDF)

S2 Fig Phylogenetic tree of *kaiC* genes in four cyanobacteria species and two purple non-sulfur bacteria strains. Two strains of *R*. *palustris* (purple non-sulfur bacteria) are shown in comparison with four species of cyanobacteria. Note that *Synechocystis sp*. PCC 6803 harbors three copies of *kaiC*, two copies of *kaiB* clustered with *kaiC* genes, and one copy of *kaiA* clustered with *kaiC*, while *Cyanothece sp*. ATCC 51142 harbors two copies of *kaiC*, two copies of *kaiB*, and one copy of *kaiA*. The *kaiA* gene is only present among cyanobacteria. The red numbers are the bootstrap values signifying the confidence of each node.
(PDF)

S3 Fig Daily patterns of nitrogen fixation in WT vs. RCKO strains. These data are replotted from Fig 1 with all three replicate cultures averaged together. Data are mean +/- S.D. (n = 6).
(PDF)

S4 Fig Lack of robust persistence of the nitrogen fixation rhythm in LL in individual cultures. Each trace represents the nitrogen fixation activity of each individual culture from the three replicates of the experiment depicted in Fig 3. **A**, WT at 30°C; **B**, RCKO at 30°C; **C**, WT at 23°C; **D**, RCKO at 23°C.
(PDF)

S5 Fig Statistical cosinor analyses of rhythmicity in LL. Modified Cosinor analyses [66] were performed to assess the rhythmicity of nitrogen fixation activities under LL conditions. The free running period (FRP) was estimated by picking the highest R^2^ value. **Upper Left Panel**: nitrogen fixation activity of the WT strain at 30°C; the highest R^2^ value is 0.2 with the corresponding FRP of about 22 h. Data were from five independent experiments. Each experiment included at least three individual cultures. **Upper Right Panel:** the nitrogen fixation activity of the WT strain at 23°C; the highest R^2^ is 0.03 with the corresponding FRP of about 22 h. Data were from 2 independent experiments, and each experiment included three individual cultures. **Lower Left Panel:** the RCKO strain at 30°C; the R^2^ was 0.0008. Data were from 2 independent experiments, and each experiment included three separate cultures.
(PDF)

S6 Fig Co-migration of native KaiC^Rp^ abundance and ATPase activity by gel filtration chromatography. After GST affinity chromatography (on glutathione-agarose resin), cleavage from GST by protease, and ion-exchange chromatography on Q Sepharose as described in Experimental Procedures, the purified native KaiC^Rp^ was chromatographed on a gel filtration column (Superdex 200 HR 10/30) and fractions were collected. The ATPase activity of each fraction was determined as described in the Experimental Procedures. **Upper Panel:** The ATPase activity co-migrates on the gel filtration column with KaiC^Rp^ abundance, indicating that the ATPase activity is attributable to the KaiC^Rp^ rather than a contaminating protein of dissimilar molecular weight. **Lower Panel:** SDS-PAGE electrophoresis of fractions 1–30 showing the KaiC^Rp^ band in fractions 17–22. Similar results were obtained with the KaiC^Rp-EQ1EQ2^ mutant protein.
(PDF)

S7 Fig Expression of *kaiC*^*Rp*^ in *S*. *elongatus* cells affects the luminescence rhythm that reports circadian gene expression. **A**, *kaiC*^*Rp*^ was overexpressed in the WT *S*. *elongatus luxAB* reporter strain AMC149 {in AMC149, the luminescence reporter *luxAB* indicates the activity of the *psbAI* promoter (Kondo et al., 1993)}. Upper panel, no IPTG induction; lower panel, IPTG (500 μM) was applied to the cultures after entrainment. Compared to the rhythm of AMC149 (blue dots), the luminescence rhythm was suppressed and the FRP was lengthened when *kaiC*^*Rp*^ was overexpressed (red dots). The P_trc_ promoter that drives *kaiC* expression is slightly leaky (Xu et al., 2003), and therefore some KaiC^Rp^ is expressed even without IPTG treatment. The traces shown are representative examples of at least six replicates. **B**, Quantification of free-running periods in AMC149 (blue) and AMC149ox*kaiC*^*Rp*^ (red) strains with and without IPTG induction. Data are mean +/- S.D. (n = 6). See S1 Text for Supplemental Methods.
(PDF)

S1 Table Bacterial strains used in this study. (PDF)

S2 Table Plasmids used in this study. (PDF)

S3 Table Primers used in this study. (PDF)

S4 Table Time series data for Fig 1. (PDF)

S5 Table Time series data for Fig 2C. (PDF)

S6 Table Time series data for Fig 3. (PDF)

S7 Table Time series data for Fig 4D. (PDF)

## References

[1] Dunlap JC Chronobiology: Biological timekeeping Sunderland, Mass.: Sinauer 2004.

[2] Ouyang Y, Andersson CR, Kondo T, Golden SS, Johnson CH. Resonating circadian clocks enhance fitness in cyanobacteria. Proc Natl Acad Sci U S A. 1998; 95: 8660–8664. 9671734 10.1073/pnas.95.15.8660 PMC21132

[3] Woelfle MA, Ouyang Y, Phanvijhitsiri K, Johnson CH. The adaptive value of circadian clocks: an experimental assessment in cyanobacteria. Curr Biol. 2004;14: 1481–1486. 15324665 10.1016/j.cub.2004.08.023

[4] Ma P, Woelfle MA, Johnson CH. An Evolutionary Fitness Enhancement Conferred by the Circadian System in Cyanobacteria. Chaos Solitons Fractals. 2013; 50: 65–74. 23626410 10.1016/j.chaos.2012.11.006 PMC3633149

[5] Johnson CH, Golden SS, Ishiura M, Kondo T. Circadian clocks in prokaryotes. Mol Microbiol. 1996; 21: 5–11. 8843429 10.1046/j.1365-2958.1996.00613.x

[6] Grobbelaar NH, Lin HY, Chow TJ. Dinitrogen-fixing endogenous rhythm in *Synechococcus* RF-1. FEMS Microbiology Letters. 1986; 37: 173–177.

[7] Kondo T, Strayer CA, Kulkarni RD, Taylor W, Ishiura M, et al Circadian rhythms in prokaryotes: luciferase as a reporter of circadian gene expression in cyanobacteria. Proc Natl Acad Sci U S A. 1993; 90: 5672–5676. 8516317 10.1073/pnas.90.12.5672 PMC46783

[8] Ito H, Mutsuda M, Murayama Y, Tomita J, Hosokawa N, et al Cyanobacterial daily life with Kai-based circadian and diurnal genome-wide transcriptional control in *Synechococcus elongatus*. Proc Natl Acad Sci U S A. 2009; 106: 14168–14173. 10.1073/pnas.0902587106 19666549 PMC2729038

[9] Smith RM, Williams SB. Circadian rhythms in gene transcription imparted by chromosome compaction in the cyanobacterium *Synechococcus elongatus*. Proc Natl Acad Sci U S A. 2006; 103: 8564–8569. 16707582 10.1073/pnas.0508696103 PMC1482530

[10] Taniguchi Y, Takai N, Katayama M, Kondo T, Oyama T. Three major output pathways from the KaiABC-based oscillator cooperate to generate robust circadian kaiBC expression in cyanobacteria. Proc Natl Acad Sci U S A. 2010; 107: 3263–3268. 10.1073/pnas.0909924107 20133618 PMC2840301

[11] Vijayan V, Zuzow R, O'Shea EK. Oscillations in supercoiling drive circadian gene expression in cyanobacteria. Proc Natl Acad Sci U S A. 2009; 106: 22564–22568. 10.1073/pnas.0912673106 20018699 PMC2799730

[12] Vijayan V, Jain IH, O'Shea EK. A high resolution map of a cyanobacterial transcriptome. Genome Biol. 2011; 12: R47 10.1186/gb-2011-12-5-r47 21612627 PMC3219970

[13] Woelfle MA, Xu Y, Qin X, Johnson CH. Circadian rhythms of superhelical status of DNA in cyanobacteria. Proc Natl Acad Sci U S A. 2007; 104: 18819–18824. 18000054 10.1073/pnas.0706069104 PMC2141860

[14] Dong G, Yang Q, Wang Q, Kim YI, Wood TL, et al Elevated ATPase activity of KaiC applies a circadian checkpoint on cell division in *Synechococcus elongatus*. Cell. 2010; 140: 529–539. 10.1016/j.cell.2009.12.042 20178745 PMC3031423

[15] Liu Y, Tsinoremas NF, Golden SS, Kondo T, Johnson CH. Circadian expression of genes involved in the purine biosynthetic pathway of the cyanobacterium *Synechococcus* sp. strain PCC 7942. Mol Microbiol. 1996; 20: 1071–1081. 8809759 10.1111/j.1365-2958.1996.tb02547.x

[16] Mori T, Binder B, Johnson CH. Circadian gating of cell division in cyanobacteria growing with average doubling times of less than 24 hours. Proc Natl Acad Sci U S A. 1996; 93: 10183–10188. 8816773 10.1073/pnas.93.19.10183 PMC38358

[17] Diamond S, Jun D, Rubin BE, Golden SS. The circadian oscillator in *Synechococcus elongatus* controls metabolite partitioning during diurnal growth. Proc Natl Acad Sci U S A. 2015; 112: E1916–1925. 10.1073/pnas.1504576112 25825710 PMC4403147

[18] Ishiura M, Kutsuna S, Aoki S, Iwasaki H, Andersson CR, et al Expression of a gene cluster kaiABC as a circadian feedback process in cyanobacteria. Science. 1998; 281: 1519–1523. 9727980 10.1126/science.281.5382.1519

[19] Nakajima M, Imai K, Ito H, Nishiwaki T, Murayama Y, et al Reconstitution of circadian oscillation of cyanobacterial KaiC phosphorylation in vitro. Science. 2005; 308: 414–415. 15831759 10.1126/science.1108451

[20] Tomita J, Nakajima M, Kondo T, Iwasaki H. No transcription-translation feedback in circadian rhythm of KaiC phosphorylation. Science. 2005; 307: 251–254. 15550625 10.1126/science.1102540

[21] Johnson CH, Stewart PL, Egli M. The cyanobacterial circadian system: from biophysics to bioevolution. Annu Rev Biophys. 2011; 40: 143–167. 10.1146/annurev-biophys-042910-155317 21332358 PMC3093959

[22] Egli M, Johnson CH. A circadian clock nanomachine that runs without transcription or translation. Curr Opin Neurobiol. 2013; 23: 732–740. 10.1016/j.conb.2013.02.012 23571120 PMC3735861

[23] Mori T, Williams DR, Byrne MO, Qin X, Egli M, et al Elucidating the ticking of an in vitro circadian clockwork. PLoS Biol. 2007; 5: e93 17388688 10.1371/journal.pbio.0050093 PMC1831719

[24] Loza-Correa M, Gomez-Valero L, Buchrieser C. Circadian clock proteins in prokaryotes: hidden rhythms? Front Microbiol. 2010; 1: 130 10.3389/fmicb.2010.00130 21687756 PMC3109361

[25] Dvornyk V, Vinogradova O, Nevo E. Origin and evolution of circadian clock genes in prokaryotes. Proc Natl Acad Sci U S A. 2003; 100: 2495–2500. 12604787 10.1073/pnas.0130099100 PMC151369

[26] Holtzendorff J, Partensky F, Mella D, Lennon JF, Hess WR, et al Genome streamlining results in loss of robustness of the circadian clock in the marine cyanobacterium *Prochlorococcus* marinus PCC 9511. J Biol Rhythms. 2008; 23: 187–199. 10.1177/0748730408316040 18487411

[27] Zinser ER, Lindell D, Johnson ZI, Futschik ME, Steglich C, et al Choreography of the transcriptome, photophysiology, and cell cycle of a minimal photoautotroph, *prochlorococcus*. PLoS One. 2009; 4: e5135 10.1371/journal.pone.0005135 19352512 PMC2663038

[28] Mullineaux CW, Stanewsky R. The rolex and the hourglass: a simplified circadian clock in *prochlorococcus*? J Bacteriol. 2009; 191: 5333–5335. 10.1128/JB.00719-09 19561127 PMC2725613

[29] Larimer FW, Chain P, Hauser L, Lamerdin J, Malfatti S, et al Complete genome sequence of the metabolically versatile photosynthetic bacterium *Rhodopseudomonas palustris*. Nat Biotechnol. 2004; 22: 55–61. 14704707 10.1038/nbt923

[30] Jiao Y, Kappler A, Croal LR, Newman DK. Isolation and characterization of a genetically tractable photoautotrophic Fe(II)-oxidizing bacterium, *Rhodopseudomonas palustris* strain TIE-1. Appl Environ Microbiol. 2005; 71: 4487–4496. 16085840 10.1128/AEM.71.8.4487-4496.2005 PMC1183355

[31] Proctor LM. Nitrogen-fixing, photosynthetic, anaerobic bacteria associated with pelagic copepods. Aquat Microb Ecol. 1997; 12: 105–113.

[32] Stal LJ. Physiological ecology of cyanobacteria in microbial mats and other communities. New Phytol. 1995: 1–32. 10.1111/j.1469-8137.1995.tb03051.x 33863161

[33] Schneegurt MA, Sherman DM, Nayar S, Sherman LA. Oscillating behavior of carbohydrate granule formation and dinitrogen fixation in the cyanobacterium Cyanothece sp. strain ATCC 51142. J Bacteriol. 1994; 176: 1586–1597. 8132452 10.1128/jb.176.6.1586-1597.1994 PMC205243

[34] Iwasaki H, Nishiwaki T, Kitayama Y, Nakajima M, Kondo T. KaiA-stimulated KaiC phosphorylation in circadian timing loops in cyanobacteria. Proc Natl Acad Sci U S A. 2002; 99: 15788–15793. 12391300 10.1073/pnas.222467299 PMC137794

[35] Xu Y, Mori T, Johnson CH. Cyanobacterial circadian clockwork: roles of KaiA, KaiB and the kaiBC promoter in regulating KaiC. Embo J. 2003; 22: 2117–2126. 12727878 10.1093/emboj/cdg168 PMC156062

[36] Qin X, Byrne M, Mori T, Zou P, Williams DR, et al Intermolecular associations determine the dynamics of the circadian KaiABC oscillator. Proc Natl Acad Sci U S A. 2010; 107: 14805–14810. 10.1073/pnas.1002119107 20679240 PMC2930409

[37] Xu Y, Mori T, Johnson CH. Circadian clock-protein expression in cyanobacteria: rhythms and phase setting. EMBO J. 2000; 19: 3349–3357. 10880447 10.1093/emboj/19.13.3349 PMC313937

[38] Terauchi K, Kitayama Y, Nishiwaki T, Miwa K, Murayama Y, et al ATPase activity of KaiC determines the basic timing for circadian clock of cyanobacteria. Proc Natl Acad Sci U S A. 2007; 104: 16377–16381. 17901204 10.1073/pnas.0706292104 PMC2042214

[39] Murakami R, Miyake A, Iwase R, Hayashi F, Uzumaki T, et al ATPase activity and its temperature compensation of the cyanobacterial clock protein KaiC. Genes Cells. 2008; 13: 387–395. 10.1111/j.1365-2443.2008.01174.x 18363969

[40] Min H, Guo H, Xiong J. Rhythmic gene expression in a purple photosynthetic bacterium *Rhodobacter sphaeroides*. FEBS Lett. 2005; 579: 808–812. 15670851 10.1016/j.febslet.2005.01.003

[41] Roenneberg T, Merrow M. Life before the clock: modeling circadian evolution. J Biol Rhythms. 2002; 17: 495–505. 12465882 10.1177/0748730402238231

[42] Troein C, Locke JC, Turner MS, Millar AJ. Weather and seasons together demand complex biological clocks. Curr Biol. 2009; 19: 1961–1964. 10.1016/j.cub.2009.09.024 19818616

[43] Leipe DD, Aravind L, Grishin NV, Koonin EV. The bacterial replicative helicase DnaB evolved from a RecA duplication. Genome Res. 2000; 10: 5–16. 10645945

[44] Bustos SA, Golden SS. Expression of the psbDII gene in *Synechococcus sp*. strain PCC 7942 requires sequences downstream of the transcription start site. J Bacteriol. 1991; 173: 7525–7533. 1938947 10.1128/jb.173.23.7525-7533.1991 PMC212519

[45] Hirakawa H, Oda Y, Phattarasukol S, Armour CD, Castle JC, et al Activity of the *Rhodopseudomonas palustris* p-coumaroyl-homoserine lactone-responsive transcription factor RpaR. J Bacteriol. 2011; 193: 2598–2607. 10.1128/JB.01479-10 21378182 PMC3133176

[46] Bose A, Newman DK. Regulation of the phototrophic iron oxidation (pio) genes in *Rhodopseudomonas palustris* TIE-1 is mediated by the global regulator, FixK. Mol Microbiol. 2011; 79: 63–75. 10.1111/j.1365-2958.2010.07430.x 21166894 PMC3050613

[47] Quandt J, Hynes MF. Versatile suicide vectors which allow direct selection for gene replacement in gram-negative bacteria. Gene. 1993; 127: 15–21. 8486283 10.1016/0378-1119(93)90611-6

[48] Simon LD, Randolph B, Irwin N, Binkowski G. Stabilization of proteins by a bacteriophage T4 gene cloned in Escherichia coli. Proc Natl Acad Sci U S A. 1983; 80: 2059–2062. 6340113 10.1073/pnas.80.7.2059 PMC393752

[49] Xu Y, Ma P, Shah P, Rokas A, Liu Y. Non-optimal codon usage is a mechanism to achieve circadian clock conditionality. Nature. 2013; 495: 116–120. 10.1038/nature11942 23417065 PMC3593822

[50] Hardy RW, Holsten RD, Jackson EK, Burns RC. The acetylene-ethylene assay for N_2_ fixation: laboratory and field evaluation. Plant Physiol. 1986; 43: 1185–1207. 10.1104/pp.43.8.1185 PMC1086994 16656902

[51] Dereeper A, Audic S, Claverie JM, Blanc G. BLAST-EXPLORER helps you building datasets for phylogenetic analysis. BMC Evol Biol. 2010; 10: 8 10.1186/1471-2148-10-8 20067610 PMC2821324

[52] Dereeper A, Guignon V, Blanc G, Audic S, Buffet S, et al Phylogeny.fr: robust phylogenetic analysis for the non-specialist. Nucleic Acids Res. 2008; 36: W465–469. 10.1093/nar/gkn180 18424797 PMC2447785

[53] Edgar RC. MUSCLE: a multiple sequence alignment method with reduced time and space complexity. BMC Bioinformatics. 2004; 5: 113 15318951 10.1186/1471-2105-5-113 PMC517706

[54] Castresana J. Selection of conserved blocks from multiple alignments for their use in phylogenetic analysis. Mol Biol Evol. 2000; 17: 540–552. 10742046 10.1093/oxfordjournals.molbev.a026334

[55] Guindon S, Gascuel O. A simple, fast, and accurate algorithm to estimate large phylogenies by maximum likelihood. Syst Biol. 2003; 52: 696–704. 14530136 10.1080/10635150390235520

[56] Chevenet F, Brun C, Banuls AL, Jacq B, Christen R. TreeDyn: towards dynamic graphics and annotations for analyses of trees. BMC Bioinformatics. 2006; 7: 439 17032440 10.1186/1471-2105-7-439 PMC1615880

[57] Anisimova M, Gascuel O. Approximate likelihood-ratio test for branches: A fast, accurate, and powerful alternative. Syst Biol. 2006; 55: 539–552. 16785212 10.1080/10635150600755453

[58] Simossis VA, Heringa J. PRALINE: a multiple sequence alignment toolbox that integrates homology-extended and secondary structure information. Nucleic Acids Res. 2005; 33: W289–294. 15980472 10.1093/nar/gki390 PMC1160151

[59] Simossis VA, Kleinjung J, Heringa J. Homology-extended sequence alignment. Nucleic Acids Res. 2005; 33: 816–824. 15699183 10.1093/nar/gki233 PMC549400

[60] Heringa J. Local weighting schemes for protein multiple sequence alignment. Comput Chem. 2002; 26: 459–477. 12144176 10.1016/s0097-8485(02)00008-6

[61] Heringa J. Computational methods for protein secondary structure prediction using multiple sequence alignments. Curr Protein Pept Sci. 2000; 1: 273–301. 12369910 10.2174/1389203003381324

[62] Xu Y, Mori T, Qin X, Yan H, Egli M, Johnson CH. Intramolecular regulation of phosphorylation status of the circadian clock protein KaiC. PLoS ONE. 2009; 4: e7509 10.1371/journal.pone.0007509 19946629 PMC2778140

[63] Kinoshita E, Kinoshita-Kikuta E, Takiyama K, Koike T. Phosphate-binding tag, new tool to visualize phosphorylated proteins. Mol Cell Proteomics. 2006; 5, 749–757. 16340016 10.1074/mcp.T500024-MCP200

[64] Lowry OH, Rosebrough NJ, Farr AL, Randall RJ. Protein measurement with the Folin phenol reagent. J Biol Chem. 1951; 193: 265–275. 14907713

[65] Sudo J, Terui J, Iwase H, Kakuno K. Assay of ATPase and Na,K-ATPase activity using high-performance liquid chromatographic determination of ADP derived from ATP. J Chromatogr B Biomed Sci Appl. 2000; 744: 19–23. 10985562 10.1016/s0378-4347(00)00218-8

[66] Kucho K, Okamoto K, Tsuchiya Y, Nomura S, Nango M, et al Global analysis of circadian expression in the cyanobacterium *Synechocystis sp*. strain PCC 6803. J Bacteriol. 2005; 187: 2190–2199. 15743968 10.1128/JB.187.6.2190-2199.2005 PMC1064041

