# Supplementary material for: Evolution of KaiC-Dependent Timekeepers: A Proto-circadian Timing Mechanism Confers Adaptive Fitness in the Purple Bacterium Rhodopseudomonas palustris
Source: PLoS Genet. 2016 Mar 16;12(3):e1005922. doi: 10.1371/journal.pgen.1005922 (PMC4794148; doi:10.1371/journal.pgen.1005922)
Supplement: S1 Text — Including: Construction of kaiCRp expression strains in S. elongatus, Measuring luminescence rhythms in cyanobacterial strains. (PDF) [file pgen.1005922.s001.pdf]

Text S1

Evolution of KaiC-dependent timekeepers: a proto-circadian timing mechanism confers adaptive fitness in the purple bacterium *Rhodopseudomonas palustris*

Peijun Ma, Tetsuya Mori, Chi Zhao, Teresa Thiel, Carl Hirschie Johnson

Supplemental Methods

Construction of *kaiC*^{Rp} expression strains in *S. elongatus*

The *kaiC*^{Rp} gene was cloned from genomic DNA of *R. palustris* TIE-1 strain and ligated with the plasmid Ptrc-NSII by *NdeI* (Xu et al., 2013). The resulting plasmid, Ptrc-NSII-kaiC^{Rp}, was transformed into the wild-type reporter strain AMC149 (Kondo et al., 1993). By transformation, the *kaiC*^{Rp} gene was incorporated into the Neutral Site II of the genome of AMC149 under the control of an IPTG-inducible promoter *trc* {P_{trc}, (Xu et al., 2013)}, resulting in the corresponding *kaiC*^{Rp} overexpression strain, AMC149ox*kaiC*^{Rp}. The *kaiC*^{Rp} overexpression strain harbors the *psbAIp::luxAB* luminescence reporter located at the Neutral Site I of AMC149 (Kondo et al., 1993; Xu et al., 2000).

Measuring luminescence rhythms in cyanobacterial strains

The *S. elongatus kaiC*^{Rp} overexpressing strain AMC149ox*kaiC*^{Rp} was cultured on BG-11 solid medium supplemented with appropriate antibiotics (Kondo et al., 1993; Xu et al., 2000). Before measuring luminescence rhythms, toothpick colonies of these strains were grown at 30°C for two LD cycles. After the cells were released to LL, the inducer IPTG was added to final concentrations of 0, 5, 10, 100, 250, 500 and 1000 μM to induce the expression of *kaiC*^{Rp}. Then the agar plates containing these strains were placed in a custom luminescence monitoring apparatus (Ishiura et al., 1998) to measure the luminescence rhythms for 5-7 d at 30°C and constant light conditions (40-50 μE $m^{-2}s^{-1}$). AMC149 colonies were also included on the agar plates as controls.

Supplemental References

Ishiura M, Kutsuna S, Aoki S, Iwasaki H, Andersson CR, Tanabe A, Golden SS, Johnson CH, and Kondo T. Expression of a gene cluster kaiABC as a circadian feedback process in cyanobacteria. Science. 1998; *281*: 1519-1523.

Kondo T, Strayer CA, Kulkarni RD, Taylor W, Ishiura M, Golden SS, and Johnson CH.Circadian rhythms in prokaryotes: luciferase as a reporter of circadian gene expression in cyanobacteria. Proc Natl Acad Sci USA. 1993; *90*: 5672-5676.

Xu Y, Mori T, and Johnson CH. Circadian clock-protein expression in cyanobacteria: rhythms and phase setting. EMBO J. 2000; *19*: 3349-3357.

Xu Y, Mori T, and Johnson CH. Cyanobacterial circadian clockwork: roles of KaiA, KaiB and the kaiBC promoter in regulating KaiC. EMBO J. 2003; *22*: 2117-2126.
