# Supplementary material for: Evolution of KaiC-Dependent Timekeepers: A Proto-circadian Timing Mechanism Confers Adaptive Fitness in the Purple Bacterium Rhodopseudomonas palustris
Source: PLoS Genet. 2016 Mar 16;12(3):e1005922. doi: 10.1371/journal.pgen.1005922 (PMC4794148; doi:10.1371/journal.pgen.1005922)
Supplement: S1 Fig — (A) Arrangement of kaiASe, kaiBSe, and kaiCSe genes in the genome of S. elongatus (separate promoters drive expression of kaiA and kaiBC transcripts) as compared with kaiCRp, kaiBRp, and a histidine kinase gene with a PAS domain in R. palustris (bioinformatic analyses suggest that a single promoter drives expression of these three genes). (B) Comparison of motifs found in kaiCRp versus kaiCSe shows the similarity of these genes. Both of the kaiC genes contain two RecA-like NTPase superfamily domains that include Walker A and Walker B motifs, catalytic EE residues, and DXXG motifs. The known phosphorylation sites of KaiCSe are located in the second domain (CII) as TST, while in KaiCRp they are TSS. The C-terminus of KaiCRp is about 50 amino acid longer than that of KaiCSe. (PDF) [file pgen.1005922.s002.pdf]

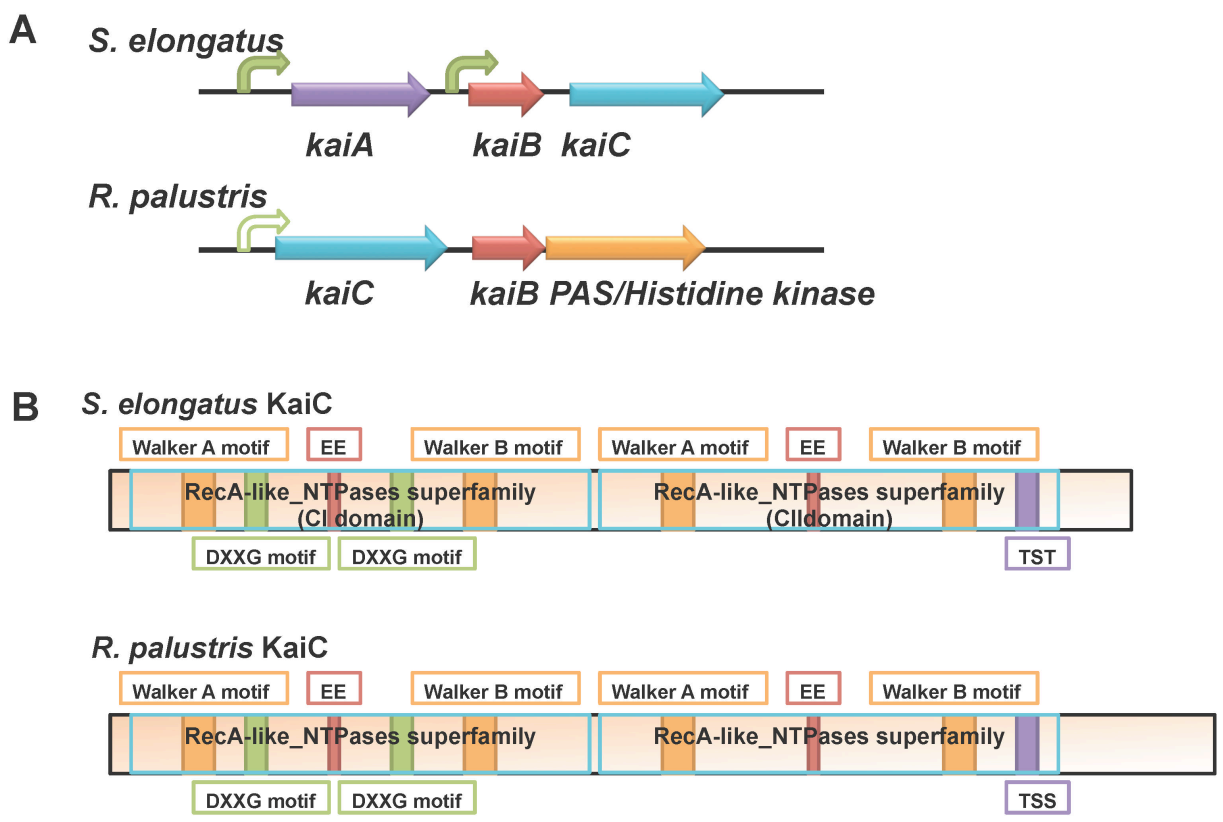


**Figure S1. Similar *kaiBC* genes of *R. palustris* and *S. elongatus*. (A)** Arrangement of *kaiA*$^{Se}$, *kaiB*$^{Se}$, and *kaiC*$^{Se}$ genes in the genome of *S. elongatus* (separate promoters drive expression of *kaiA* and *kaiBC* transcripts) as compared with *kaiC*$^{Rp}$, *kaiB*$^{Rp}$, and a histidine kinase gene with a PAS domain in *R. palustris* (bioinformatic analyses suggest that a single promoter drives expression of these three genes). **(B)** Comparison of motifs found in *kaiC*$^{Rp}$ versus *kaiC*$^{Se}$ shows the similarity of these genes. Both of the *kaiC* genes contain two RecA-like NTPase superfamily domains that include Walker A and Walker B motifs, catalytic EE residues, and DXXG motifs. The known phosphorylation sites of KaiC$^{Se}$ are located in the second domain (CII) as TST, while in KaiC$^{Rp}$ they are TSS. The C-terminus of KaiC$^{Rp}$ is about 50 amino acid longer than that of KaiC$^{Se}$.
