# Supplementary material for: Evolution of KaiC-Dependent Timekeepers: A Proto-circadian Timing Mechanism Confers Adaptive Fitness in the Purple Bacterium Rhodopseudomonas palustris
Source: PLoS Genet. 2016 Mar 16;12(3):e1005922. doi: 10.1371/journal.pgen.1005922 (PMC4794148; doi:10.1371/journal.pgen.1005922)
Supplement: S2 Fig — Two strains of R. palustris (purple non-sulfur bacteria) are shown in comparison with four species of cyanobacteria. Note that Synechocystis sp. PCC 6803 harbors three copies of kaiC, two copies of kaiB clustered with kaiC genes, and one copy of kaiA clustered with kaiC, while Cyanothece sp. ATCC 51142 harbors two copies of kaiC, two copies of kaiB, and one copy of kaiA. The kaiA gene is only present among cyanobacteria. The red numbers are the bootstrap values signifying the confidence of each node. (PDF) [file pgen.1005922.s003.pdf]

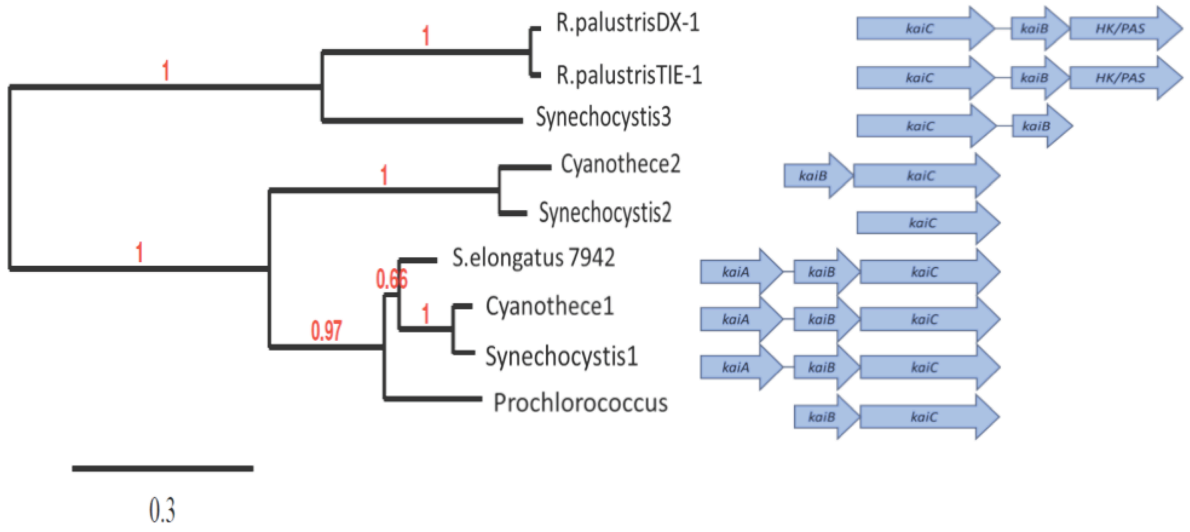

Figure S2. Phylogenetic tree of *kaiC* genes in four cyanobacteria species and two purple non-sulfur bacteria strains. Two strains of *R. palustris* (purple non-sulfur bacteria) are shown in comparison with four species of cyanobacteria. Note that *Synechocystis sp.* PCC 6803 harbors three copies of *kaiC*, two copies of *kaiB* clustered with *kaiC* genes, and one copy of *kaiA* clustered with *kaiC*, while *Cyanothece sp.* ATCC 51142 harbors two copies of *kaiC*, two copies of *kaiB*, and one copy of *kaiA*. The *kaiA* gene is only present among cyanobacteria. The red numbers are the bootstrap values signifying the confidence of each node.
