# Supplementary material for: Evolution of KaiC-Dependent Timekeepers: A Proto-circadian Timing Mechanism Confers Adaptive Fitness in the Purple Bacterium Rhodopseudomonas palustris
Source: PLoS Genet. 2016 Mar 16;12(3):e1005922. doi: 10.1371/journal.pgen.1005922 (PMC4794148; doi:10.1371/journal.pgen.1005922)
Supplement: S3 Fig — These data are replotted from Fig 1 with all three replicate cultures averaged together. Data are mean +/- S.D. (n = 6). (PDF) [file pgen.1005922.s004.pdf]

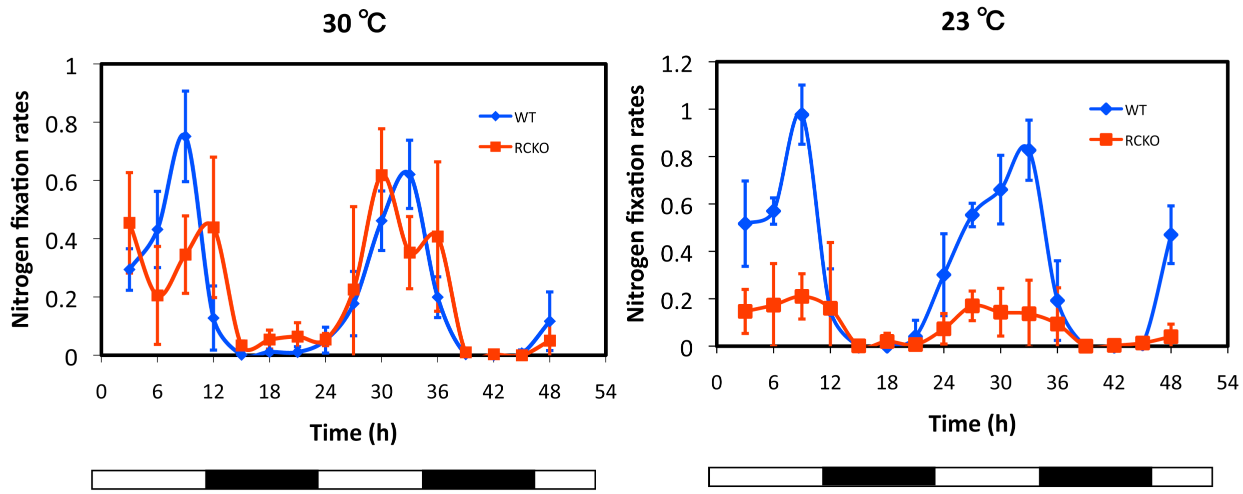

Figure S3. Daily patterns of nitrogen fixation in WT vs. RCKO strains. These data are replotted from Fig. 1 with all three replicate cultures averaged together. Data are mean +/- S.D. (n = 6).
