# Supplementary material for: Evolution of KaiC-Dependent Timekeepers: A Proto-circadian Timing Mechanism Confers Adaptive Fitness in the Purple Bacterium Rhodopseudomonas palustris
Source: PLoS Genet. 2016 Mar 16;12(3):e1005922. doi: 10.1371/journal.pgen.1005922 (PMC4794148; doi:10.1371/journal.pgen.1005922)
Supplement: S4 Fig — Each trace represents the nitrogen fixation activity of each individual culture from the three replicates of the experiment depicted in Fig 3. A, WT at 30°C; B, RCKO at 30°C; C, WT at 23°C; D, RCKO at 23°C. (PDF) [file pgen.1005922.s005.pdf]

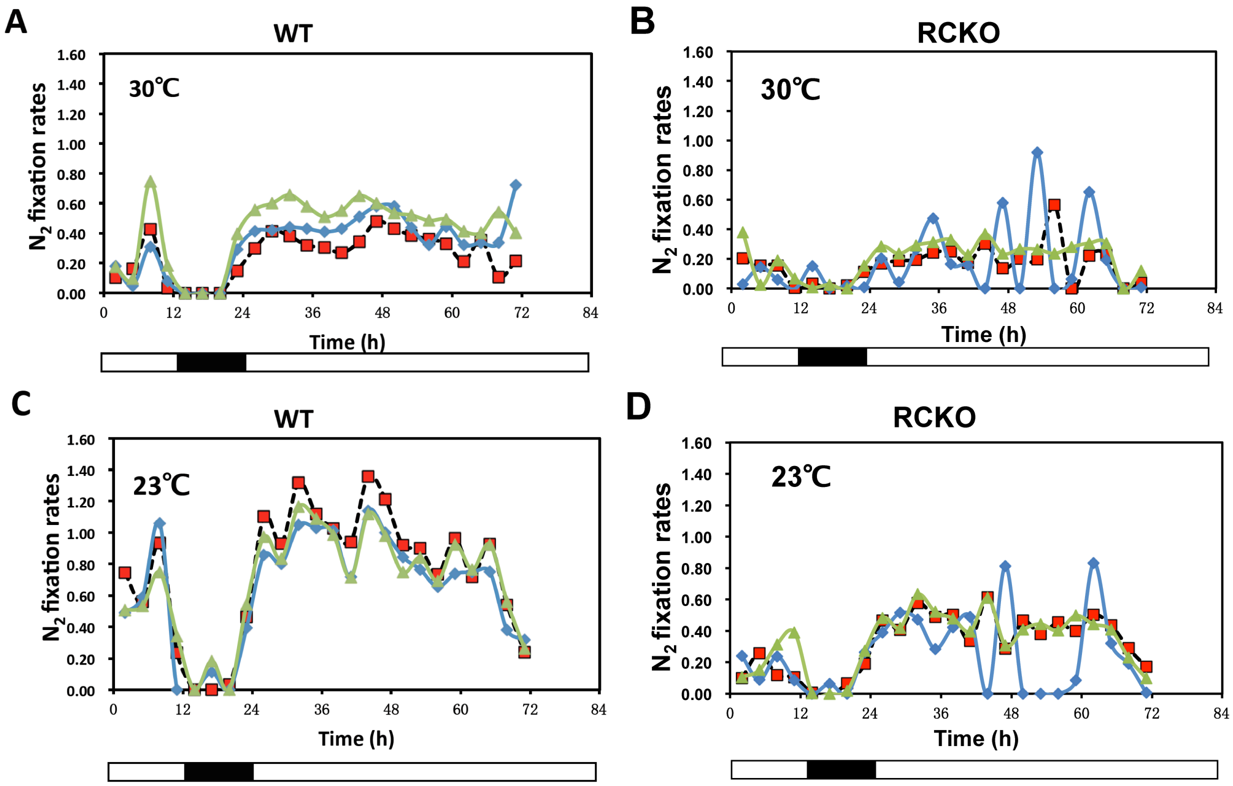

Figure S4. Lack of robust persistence of the nitrogen fixation rhythm in LL in individual cultures. Each trace represents the nitrogen fixation activity of each individual culture from the three replicates of the experiment depicted in Figure 3. **A**, WT at 30°C; **B**, RCKO at 30°C; **C**, WT at 23°C; **D**, RCKO at 23°C.
