# Supplementary material for: Evolution of KaiC-Dependent Timekeepers: A Proto-circadian Timing Mechanism Confers Adaptive Fitness in the Purple Bacterium Rhodopseudomonas palustris
Source: PLoS Genet. 2016 Mar 16;12(3):e1005922. doi: 10.1371/journal.pgen.1005922 (PMC4794148; doi:10.1371/journal.pgen.1005922)
Supplement: S5 Fig — Modified Cosinor analyses [66] were performed to assess the rhythmicity of nitrogen fixation activities under LL conditions. The free running period (FRP) was estimated by picking the highest R2 value. Upper Left Panel: nitrogen fixation activity of the WT strain at 30°C; the highest R2 value is 0.2 with the corresponding FRP of about 22 h. Data were from five independent experiments. Each experiment included at least three individual cultures. Upper Right Panel: the nitrogen fixation activity of the WT strain at 23°C; the highest R2 is 0.03 with the corresponding FRP of about 22 h. Data were from 2 independent experiments, and each experiment included three individual cultures. Lower Left Panel: the RCKO strain at 30°C; the R2 was 0.0008. Data were from 2 independent experiments, and each experiment included three separate cultures. (PDF) [file pgen.1005922.s006.pdf]

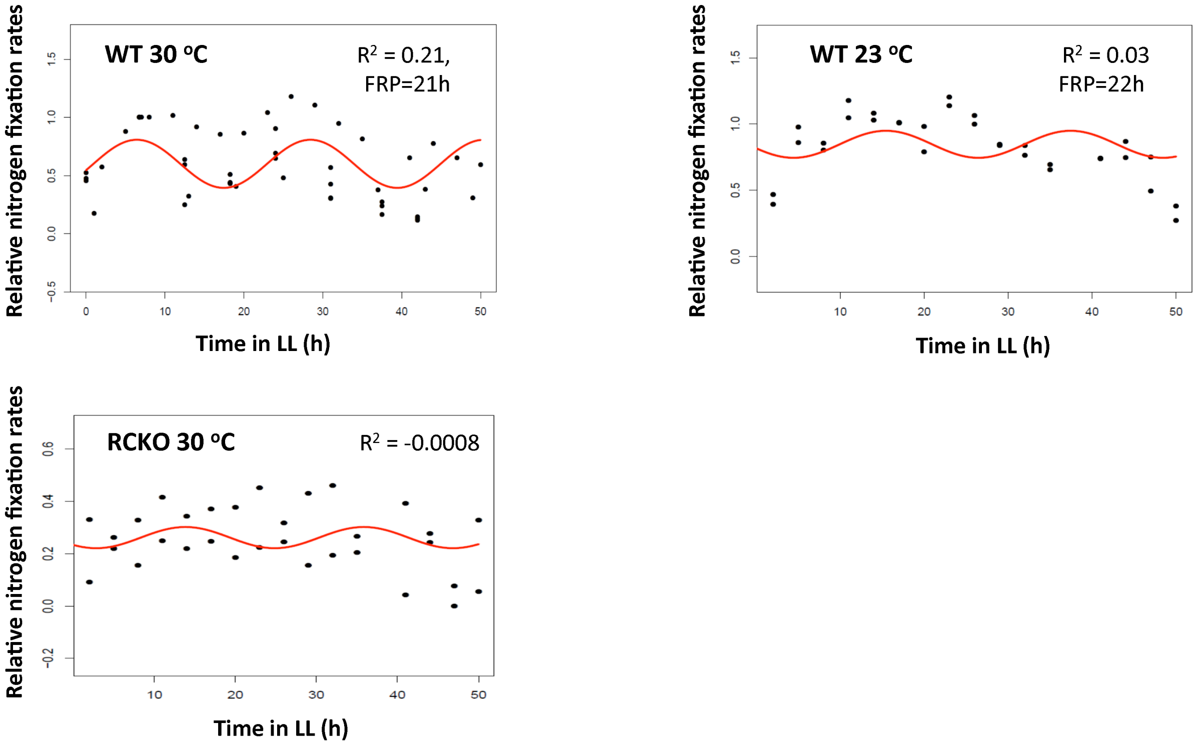

Figure S5. Statistical cosinor analyses of rhythmicity in LL. Modified Cosinor analyses [65] were performed to assess the rhythmicity of nitrogen fixation activities under LL conditions. The free running period (FRP) was estimated by picking the highest R^2 value. **Upper Left Panel**: nitrogen fixation activity of the WT strain at 30°C; the highest R^2 value is 0.2 with the corresponding FRP of about 22 h. Data were from five independent experiments. Each experiment included at least three individual cultures. **Upper Right Panel:** the nitrogen fixation activity of the WT strain at 23°C; the highest R^2 is 0.03 with the corresponding FRP of about 22 h. Data were from 2 independent experiments, and each experiment included three individual cultures. **Lower Left Panel:** the RCKO strain at 30°C; the R^2 was 0.0008. Data were from 2 independent experiments, and each experiment included three separate cultures.
