# Supplementary material for: Evolution of KaiC-Dependent Timekeepers: A Proto-circadian Timing Mechanism Confers Adaptive Fitness in the Purple Bacterium Rhodopseudomonas palustris
Source: PLoS Genet. 2016 Mar 16;12(3):e1005922. doi: 10.1371/journal.pgen.1005922 (PMC4794148; doi:10.1371/journal.pgen.1005922)
Supplement: S6 Fig — After GST affinity chromatography (on glutathione-agarose resin), cleavage from GST by protease, and ion-exchange chromatography on Q Sepharose as described in Experimental Procedures, the purified native KaiCRp was chromatographed on a gel filtration column (Superdex 200 HR 10/30) and fractions were collected. The ATPase activity of each fraction was determined as described in the Experimental Procedures. Upper Panel: The ATPase activity co-migrates on the gel filtration column with KaiCRp abundance, indicating that the ATPase activity is attributable to the KaiCRp rather than a contaminating protein of dissimilar molecular weight. Lower Panel: SDS-PAGE electrophoresis of fractions 1–30 showing the KaiCRp band in fractions 17–22. Similar results were obtained with the KaiCRp-EQ1EQ2 mutant protein. (PDF) [file pgen.1005922.s007.pdf]

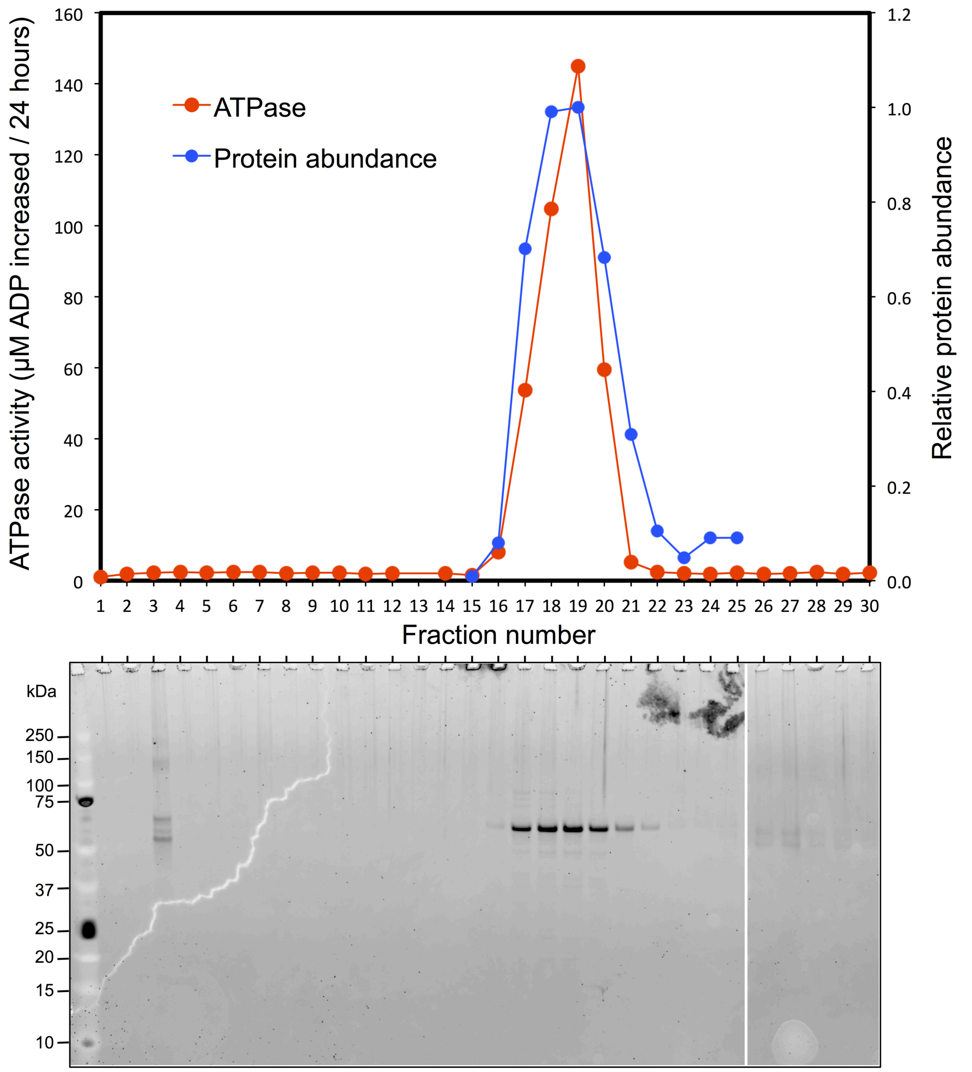

Figure S6. Co-migration of native KaiC^{Rp} abundance and ATPase activity by gel filtration chromatography. After GST affinity chromatography (on glutathione-agarose resin), cleavage from GST by protease, and ion-exchange chromatography on Q Sepharose as described in Experimental Procedures, the purified native KaiC^{Rp} was chromatographed on a gel filtration column (Superdex 200 HR 10/30) and fractions were collected. The ATPase activity of each fraction was determined as described in the Experimental Procedures. **Upper Panel:** The ATPase activity co-migrates on the gel filtration column with KaiC^{Rp} abundance, indicating that the ATPase activity is attributable to the KaiC^{Rp} rather than a contaminating protein of dissimilar molecular weight. **Lower Panel:** SDS-PAGE electrophoresis of fractions 1-30 showing the KaiC^{Rp} band in fractions 17-22. Similar results were obtained with the KaiC$^{Rp\text{-}EQ1EQ2}$ mutant protein.
