# Supplementary material for: Evolution of KaiC-Dependent Timekeepers: A Proto-circadian Timing Mechanism Confers Adaptive Fitness in the Purple Bacterium Rhodopseudomonas palustris
Source: PLoS Genet. 2016 Mar 16;12(3):e1005922. doi: 10.1371/journal.pgen.1005922 (PMC4794148; doi:10.1371/journal.pgen.1005922)
Supplement: S7 Fig — A, kaiCRp was overexpressed in the WT S. elongatus luxAB reporter strain AMC149 {in AMC149, the luminescence reporter luxAB indicates the activity of the psbAI promoter (Kondo et al., 1993)}. Upper panel, no IPTG induction; lower panel, IPTG (500 μM) was applied to the cultures after entrainment. Compared to the rhythm of AMC149 (blue dots), the luminescence rhythm was suppressed and the FRP was lengthened when kaiCRp was overexpressed (red dots). The Ptrc promoter that drives kaiC expression is slightly leaky (Xu et al., 2003), and therefore some KaiCRp is expressed even without IPTG treatment. The traces shown are representative examples of at least six replicates. B, Quantification of free-running periods in AMC149 (blue) and AMC149oxkaiCRp (red) strains with and without IPTG induction. Data are mean +/- S.D. (n = 6). See S1 Text for Supplemental Methods. (PDF) [file pgen.1005922.s008.pdf]

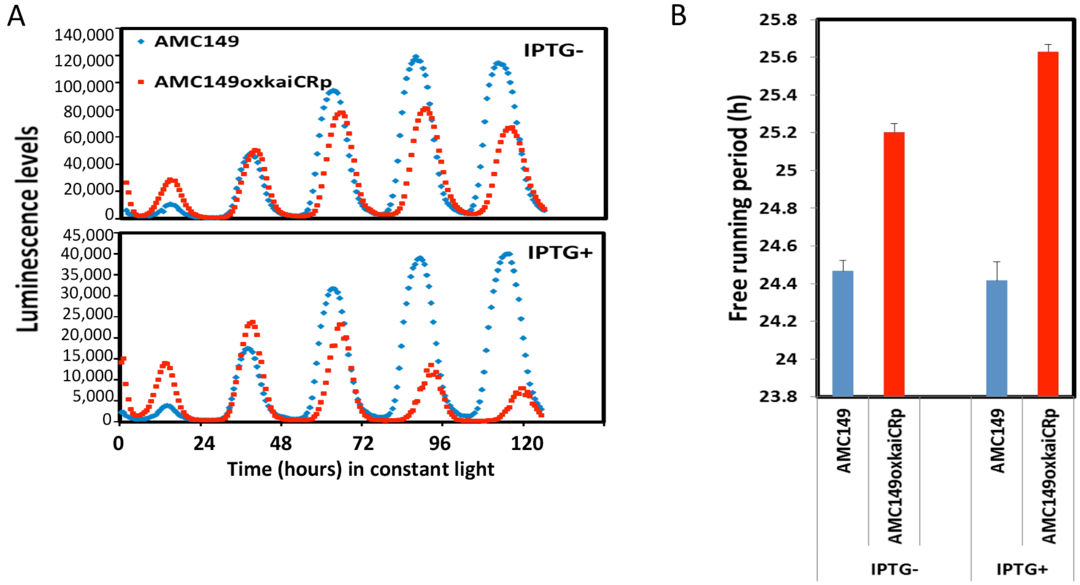


**Figure S7. Expression of *kaiC$^{Rp}$* in *S. elongatus* cells affects the luminescence rhythm that reports circadian gene expression. A**, *kaiC$^{Rp}$* was overexpressed in the WT *S. elongatus luxAB* reporter strain AMC149 {in AMC149, the luminescence reporter *luxAB* indicates the activity of the *psbAI* promoter (Kondo et al., 1993)}. Upper panel, no IPTG induction; lower panel, IPTG (500 μM) was applied to the cultures after entrainment. Compared to the rhythm of AMC149 (blue dots), the luminescence rhythm was suppressed and the FRP was lengthened when *kaiC$^{Rp}$* was overexpressed (red dots). The $P_{trc}$ promoter that drives *kaiC* expression is slightly leaky (Xu et al., 2003), and therefore some KaiC$^{Rp}$ is expressed even without IPTG treatment. The traces shown are representative examples of at least six replicates. **B**, Quantification of free-running periods in AMC149 (blue) and AMC149ox*kaiC$^{Rp}$* (red) strains with and without IPTG induction. Data are mean +/- S.D. (n = 6). See Text S1 for Methods.
