# Supplementary material for: Evolution of KaiC-Dependent Timekeepers: A Proto-circadian Timing Mechanism Confers Adaptive Fitness in the Purple Bacterium Rhodopseudomonas palustris
Source: PLoS Genet. 2016 Mar 16;12(3):e1005922. doi: 10.1371/journal.pgen.1005922 (PMC4794148; doi:10.1371/journal.pgen.1005922)
Supplement: S1 Table — (PDF) [file pgen.1005922.s009.pdf]

Table S1. Bacterial strains used in this study

Strain	Genotype and use	Reference
E.Coli S17-1	A conjugative donor strain [*thi pro hdsR hdsM+ recA*; chromosomal insertion of RP4-2 (Tc::Mu Km::Tn7)].	[45, 48]
R. palustris TIE-1	Served as the wild type strain of *R. palustris* TIE-1.	[30]
RCKO	$kaiC^{Rp}$ knockout strain (1674bp deleted from $kaiC^{Rp}$ gene in *R. palustris* TIE-1)	This study
RCKO-FLAG$kaiC^{Rp}$	A FLAG-tagged $kaiC^{Rp}$ ectopically inserted into the genome of RCKO.	This study
RCKO-HA$kaiC^{Rp}$	A HA-tagged $kaiC^{Rp}$ ectopically inserted into the genome of RCKO.	This study
