# Supplementary material for: Evolution of KaiC-Dependent Timekeepers: A Proto-circadian Timing Mechanism Confers Adaptive Fitness in the Purple Bacterium Rhodopseudomonas palustris
Source: PLoS Genet. 2016 Mar 16;12(3):e1005922. doi: 10.1371/journal.pgen.1005922 (PMC4794148; doi:10.1371/journal.pgen.1005922)
Supplement: S2 Table — (PDF) [file pgen.1005922.s010.pdf]

Table S2. Plasmids used in this study

Plasmid	Description	Reference
pJQ200KS	Mobilizable suicide vector; *sacB*, Gm^r	[45, 47]
pJQ200KS - RCKO	Overlap extension PCR was used to amplify homologous regions of 1-kb upstream and 1-kb downstream of the *kaiC*^{Rp} locus, then the 2-kb fragment was ligated with Pjq200ks by *BamHI* and *XbaI*. This plasmid was used to delete the *kaiC*^{Rp} gene in the wild-type *R. palustris.* Gm^r	This study
pJQ200KS -Insert	Overlap extension PCR was used to amplify the 2 kb region surrounding the *glmUSX-recG* locus of *R. palustris* TIE-1, a NcoI site was incorporated into the middle of this PCR fragment which was ligated to pJQ- 200KS by *Sph*I and *Sma*I, as described by Bose and Newman. This plasmid was used to restore tagged *kaiC*^{Rp} gene into the RCKO strain. Gm^r	[46]<br>This study
pJQ200KS – Insert-PkaiC^{Rp}	A 469-bp region upstream of *kaiC*^{Rp} gene locus in *R. palustris* was amplified as the promoter region of *kaiC*^{Rp} gene. This PCR fragment was ligated to the *NcoI* site of pJQ200KS – Insert. A *NdeI* and a *XbaI* sites were incorporated to the downstream of the promoter region. Gm^r	This study
pJQ200KS – Insert - PkaiC^{Rp} - FLAGkaiC^{Rp}	The *kaiC*^{Rp} gene was amplified from the wild type *R. palustris*. A FLAG tag was incorporated into the N-terminus. This PCR fragment was ligated to the downstream of promoter region of the *kaiC*^{Rp} gene in pJQ200KS – Insert-PkaiC^{Rp}. This construct was used to restore a FLAG-tagged *kaiC*^{Rp} gene to the RCKO strain. Gm^r	This study
pJQ200KS – Insert - PkaiC^{Rp} - HAkaiC^{Rp}	The *kaiC*^{Rp} gene was amplified from the wild type *R. palustris*. A HA tag was incorporated into the C-terminus. This PCR fragment was ligated to the downstream of promoter region of the *kaiC*^{Rp} gene in pJQ200KS – Insert-PkaiC^{Rp}. This construct was used to restore a HA-tagged *kaiC*^{Rp} gene to the RCKO strain. Gm^r	This study
