# Supplementary material for: Evolution of KaiC-Dependent Timekeepers: A Proto-circadian Timing Mechanism Confers Adaptive Fitness in the Purple Bacterium Rhodopseudomonas palustris
Source: PLoS Genet. 2016 Mar 16;12(3):e1005922. doi: 10.1371/journal.pgen.1005922 (PMC4794148; doi:10.1371/journal.pgen.1005922)
Supplement: S3 Table — (PDF) [file pgen.1005922.s011.pdf]

Table S3. Primers used in this study

Primer	Sequence	Description/ Reference
CupF	ATAGGATCCACAGCTCGCCTCGCGGACCGGAA	1-kb upstream of *kaiC*^{Rp} gene
CupR56	TATGGCAATCGTGTGGATGGAGTCCGCCATCGCACC ACAGG	1-kb upstream of *kaiC*^{Rp} gene
CdownF56	TCCATCCACACGATTGCCATAACGGACTAGGGGACG ACGAT	1-kb downstream of *kaiC*^{Rp} gene
CdownR	AAATCTAGACGCGCTGCGACAGATCGACCAGG	1-kb downstream of *kaiC*^{Rp} gene
TIE-1upfor	GGCGCGCCGCATGCCACACCGGCAGGTTGTTGATG GCTG	[46]
TIE-1fusionrev(new)	CGGGTTAGTTACCACGCGTCATTACTAGTTCGCGACC ATGGCTACCCGACCTTGTCCGGCGCCTTTTC	[46]
TIE-1dnrev	ACTAGTCCCGGGCGAGATCGATTTTCTGGTCGGCAC	[46]
TIE-1fusionfor(new)	GAAAAGGCGCCGGACAAGGTCGGGTAGCCATGGTC GCGAACTAGTAATGACGCGTGGTAACTAACCCG	[46]
R10(PkaiF)	GCGGCTAGCCCATGGCGTCACGTCGCGCTTTGC	promoter region of *kaiC*^{Rp} gene
R30(PrkaicR2)	CTGACCATGGTCTAGACATATGCGCACCACAGGTCG TTTTCTGA	Promoter region of *kaiC*^{Rp} gene
RCup	ATGCAACATATGGCGGACGGCATATC	Forward primer of *kaiC*^{Rp} gene
RCdown	ATGCAATCTAGACTAGTCCGTGTCATCGGC	Reverse primer of *kaiC*^{Rp} gene
FlagCup	ATGCAACATATGGACTATAAGGACGACGACGACAAG GCGGACGGCATATC	Forward primer of *kaiC*^{Rp} gene
FlagCdown	ATGCAATCTAGACTACTTGTCGTCGTCGTCCTTATAG TCGTCCGTGTCATCGGC	Reverse primer of

		kaiC^{Rp} gene
RCNdeF	ATACATATGagcATGGCGGACGGCATATC	Forward primer of *kaiC*^{Rp} gene
RCNdeR	ATACATATGagCTAGTCCGTGTCATCGGCCG	Reverse primer of *kaiC*^{Rp} gene
clxpF	GGAGATCTGCAAGGTTCTCG	qRT-PCR primer-clxp-internal control
clxpR	GATGTTCGACTTCGCCAGTT	qRT-PCR primer-clxp-internal control
RkaiCF	CCGAAGTCGGTGACTACGAT	qRT-PCR primer-kaiC^{Rp}
RkaiCR	GAACAGGCTCTCGATGGTGT	qRT-PCR primer-kaiC^{Rp}
RkaiBF	CGGCAAGTACAAGGTCGAAG	qRT-PCR primer-kaiB^{Rp}
RkaiBR	CAGGTCGCCGATGATCTTAC	qRT-PCR primer-kaiB^{Rp}
