# Supplementary material for: Evolution of KaiC-Dependent Timekeepers: A Proto-circadian Timing Mechanism Confers Adaptive Fitness in the Purple Bacterium Rhodopseudomonas palustris
Source: PLoS Genet. 2016 Mar 16;12(3):e1005922. doi: 10.1371/journal.pgen.1005922 (PMC4794148; doi:10.1371/journal.pgen.1005922)
Supplement: S4 Table — (PDF) [file pgen.1005922.s012.pdf]

Table S4. Time series data for Figure 1

30°C

Time	WT1	WT2	WT3	RCKO1	RCKO2	RCKO3
3	0.32	0.35	0.21	0.40	0.31	0.65
6	0.40	0.58	0.32	0.07	0.39	0.15
9	0.80	0.88	0.58	0.37	0.20	0.46
12	0.14	0.01	0.23	0.64	0.51	0.17
15	0.01	0.00	0.00	0.05	0.02	0.03
18	0.00	0.01	0.02	0.04	0.09	0.03
21	0.03	0.00	0.00	0.10	0.01	0.08
24	0.04	0.01	0.10	0.03	0.06	0.07
27	0.05	0.21	0.27	0.55	0.00	0.13
30	0.57	0.44	0.37	0.67	0.44	0.75
33	0.63	0.73	0.50	0.21	0.45	0.39
36	0.21	0.12	0.26	0.62	0.12	0.48
39	0.01	0.00	0.00	0.00	0.01	0.02
42	0.00	0.00	0.00	0.00	0.01	0.00
45	0.00	0.02	0.00	0.00	0.00	0.00
48	0.01	0.13	0.21	0.02	0.13	0.00

23°

Time	WT1	WT2	WT3	RCKO1	RCKO2	RCKO3
3	0.34	0.51	0.70	0.12	0.07	0.25
6	0.56	0.52	0.63	0.35	0.00	0.17
9	0.92	0.89	1.12	0.12	0.20	0.31
12	0.00	0.12	0.34	0.00	0.00	0.48
15	0.00	0.00	0.00	0.00	0.00	0.00
18	0.00	0.00	0.00	0.00	0.06	0.00
21	0.12	0.00	0.00	0.00	0.00	0.02
24	0.46	0.12	0.32	0.12	0.10	0.00
27	0.61	0.53	0.52	0.24	0.12	0.15
30	0.82	0.62	0.54	0.13	0.05	0.25
33	0.90	0.90	0.68	0.05	0.06	0.30
36	0.27	0.00	0.31	0.00	0.01	0.27
39	0.00	0.00	0.00	0.00	0.00	0.00
42	0.00	0.00	0.00	0.00	0.01	0.00
45	0.00	0.03	0.00	0.03	0.01	0.00
48	0.39	0.41	0.61	0.00	0.10	0.02
