# Supplementary material for: Evolution of KaiC-Dependent Timekeepers: A Proto-circadian Timing Mechanism Confers Adaptive Fitness in the Purple Bacterium Rhodopseudomonas palustris
Source: PLoS Genet. 2016 Mar 16;12(3):e1005922. doi: 10.1371/journal.pgen.1005922 (PMC4794148; doi:10.1371/journal.pgen.1005922)
Supplement: S5 Table — (PDF) [file pgen.1005922.s013.pdf]

Table S5. Time series data for Figure 2C

Time	RCKO+kaiCRp 1	RCKO+kaiCRp 2	RCKO+kaiCRp 3
2	0.00	0.00	0.05
5	0.18	0.10	0.17
8	0.24	0.13	0.26
11	0.00	0.01	0.07
14	0.00	0.02	0.00
17	0.00	0.00	0.00
20	0.00	0.01	0.03
23	0.00	0.02	0.04
