# Supplementary material for: Evolution of KaiC-Dependent Timekeepers: A Proto-circadian Timing Mechanism Confers Adaptive Fitness in the Purple Bacterium Rhodopseudomonas palustris
Source: PLoS Genet. 2016 Mar 16;12(3):e1005922. doi: 10.1371/journal.pgen.1005922 (PMC4794148; doi:10.1371/journal.pgen.1005922)
Supplement: S6 Table — (PDF) [file pgen.1005922.s014.pdf]

Table S6. Time series data for Figure 3

30°C

Time	WT1	WT2	WT3	RCKO1	RCKO2	RCKO3	Average Time	WT	RCKO
2	0.10	0.18	0.18	0.46	0.55	0.49	2	0.15	0.50
5	0.16	0.05	0.10	0.67	0.11	0.29	5	0.10	0.35
8	0.43	0.31	0.75	0.00	0.30	0.46	8	0.49	0.25
11	0.03	0.08	0.18	0.09	0.60	0.00	11	0.10	0.23
14	0.00	0.00	0.00	0.00	0.00	0.03	14	0.00	0.01
17	0.00	0.00	0.00	0.01	0.00	0.00	17	0.00	0.00
20	0.00	0.00	0.00	0.00	0.16	0.13	20	0.00	0.10
23	0.15	0.29	0.39	0.34	0.43	0.22	23	0.28	0.33
26	0.30	0.41	0.56	0.21	0.03	0.55	26	0.42	0.26
29	0.41	0.42	0.60	0.08	0.27	0.63	29	0.48	0.33
32	0.38	0.44	0.66	0.18	0.37	0.69	32	0.49	0.42
35	0.32	0.43	0.58	0.22	0.40	0.04	35	0.44	0.22
38	0.31	0.41	0.51	0.30	0.42	0.39	38	0.41	0.37
41	0.27	0.43	0.55	0.56	0.26	0.32	41	0.42	0.38
44	0.34	0.51	0.65	0.46	0.48	0.42	44	0.50	0.45
47	0.48	0.58	0.60	0.00	0.37	0.36	47	0.55	0.25
50	0.43	0.58	0.54	0.27	0.36	0.67	50	0.52	0.43
53	0.39	0.44	0.52	0.28	0.25	0.05	53	0.45	0.19
56	0.36	0.32	0.48	0.35	0.17	0.09	56	0.39	0.20
59	0.33	0.45	0.49	0.30	0.61	0.06	59	0.32	0.04
62	0.21	0.32	0.41	0.02	0.00	0.11	62	0.36	0.28
65	0.35	0.34	0.40	0.16	0.25	0.41	65	0.33	0.08
68	0.11	0.34	0.54	0.10	0.08	0.05	68	0.31	0.33

23°C

Time	WT1	WT2	WT3	RCKO1	RCKO2	RCKO3	Average Time	WT	RCKO
2	0.75	0.49	0.51	0.10	0.24	0.11	2	0.58	0.15
5	0.56	0.59	0.53	0.26	0.09	0.15	5	0.56	0.16
8	0.93	1.06	0.75	0.12	0.24	0.31	8	0.91	0.22
11	0.23	0.00	0.34	0.10	0.09	0.39	11	0.19	0.19
14	0.00	0.00	0.00	0.00	0.00	0.00	14	0.00	0.00
17	0.00	0.11	0.18	0.00	0.06	0.00	17	0.10	0.02
20	0.03	0.00	0.00	0.07	0.00	0.02	20	0.01	0.03
23	0.46	0.39	0.54	0.19	0.26	0.28	23	0.47	0.24
26	1.10	0.86	0.97	0.47	0.39	0.48	26	0.98	0.45
29	0.93	0.80	0.83	0.41	0.51	0.42	29	0.85	0.45
32	1.32	1.05	1.17	0.58	0.47	0.63	32	1.18	0.56
35	1.12	1.03	1.09	0.49	0.29	0.52	35	1.08	0.43
38	1.02	1.01	0.99	0.50	0.42	0.48	38	1.01	0.47
41	0.94	0.72	0.72	0.34	0.49	0.40	41	0.79	0.41
44	1.36	1.14	1.12	0.61	0.00	0.61	44	1.20	0.41
47	1.21	1.00	0.98	0.29	0.81	0.31	47	1.06	0.47
50	0.92	0.85	0.75	0.46	0.00	0.41	50	0.84	0.29
53	0.90	0.77	0.84	0.38	0.00	0.44	53	0.84	0.27
56	0.73	0.65	0.69	0.45	0.00	0.40	56	0.69	0.29
59	0.96	0.74	0.92	0.40	0.09	0.50	59	0.74	0.59
62	0.72	0.75	0.77	0.50	0.83	0.44	62	0.87	0.39
65	0.93	0.75	0.92	0.44	0.32	0.41	65	0.49	0.24
68	0.54	0.38	0.56	0.29	0.19	0.23	68	0.27	0.09
