# Supplementary material for: Evolution of KaiC-Dependent Timekeepers: A Proto-circadian Timing Mechanism Confers Adaptive Fitness in the Purple Bacterium Rhodopseudomonas palustris
Source: PLoS Genet. 2016 Mar 16;12(3):e1005922. doi: 10.1371/journal.pgen.1005922 (PMC4794148; doi:10.1371/journal.pgen.1005922)
Supplement: S7 Table — (PDF) [file pgen.1005922.s015.pdf]

Table S7. Time series data for Figure 4D

#	Hours in LL	P-KaiC/total KaiC
1	-24	0.660
2	-18	0.932
3	-12	0.938
4	-6	0.912
5	0	0.615
6	6	0.833
7	12	0.771
8	18	0.814
9	24	0.819
10	30	0.781
11	36	0.832
12	42	0.839
13	48	0.914
